# Zwitterionic and biradicaloid heteroatomic cyclopentane derivatives containing different group 15 elements†
†Electronic supplementary information (ESI) available: Additional experimental details, full characterization of all compounds and computational details. CCDC 1421413–1421416. For ESI and crystallographic data in CIF or other electronic format see DOI: 10.1039/c5sc03515e


**DOI:** 10.1039/c5sc03515e

**Published:** 2015-10-27

**Authors:** Alexander Hinz, Axel Schulz, Alexander Villinger

**Affiliations:** a Institut für Chemie , Universität Rostock , Albert-Einstein-Str. 3a , 18059 Rostock , Germany . Email: axel.schulz@uni-rostock.de; b Abteilung Materialdesign , Leibniz-Institut für Katalyse e.V. an der Universität Rostock , Albert-Einstein-Str. 29a , 18059 Rostock , Germany

## Abstract

The formal cyclopentane-1,3-diyl derivatives [E^1^(μ-NTer)_2_({E^2^C} = NDmp)] (Ter = 2,6-dimesityl-phenyl, Dmp = 2,6-dimethylphenyl) were prepared by 1,1-insertion of CNDmp into the N–E^2^ bond of [E^1^(μ-NTer)_2_E^2^] (E^1^ = N, P; E^2^ = P, As). The insertion does not occur for E^1^ = E^2^ = As or E^2^ = Sb. Dependent on the choice of formal radical centres E, either a biradicaloid or a zwitterion was obtained. The biradicaloid features a P and an As radical center and its biradical character was established by computations as well as characteristic reactivity with respect to the formation of a housane derivative and the activation of molecules bearing multiple bonds, which was demonstrated using the example of PC^*t*^Bu. In contrast, the formally N,As- and N,P-centered biradicaloids are better regarded as zwitterionic species in accord with computations and diminished reactivity, as neither housane formation nor activation of multiple bonds could be observed.

## Abstract 2

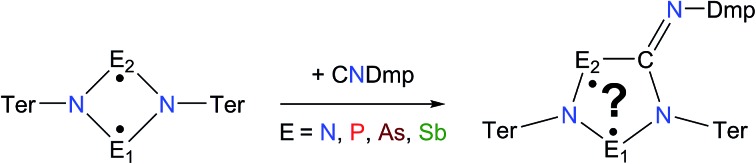
The formal cyclopentane-1,3-diyl derivatives [E^1^(μ-NTer)_2_({E^2^C} = NDmp)] (Ter = 2,6-dimesityl-phenyl, Dmp = 2,6-dimethylphenyl) were prepared by 1,1-insertion of CNDmp into the N–E^2^ bond of [E^1^(μ-NTer)_2_E^2^] (E^1^ = N, P; E^2^ = P, As).

## Introduction

**Scheme 1:**
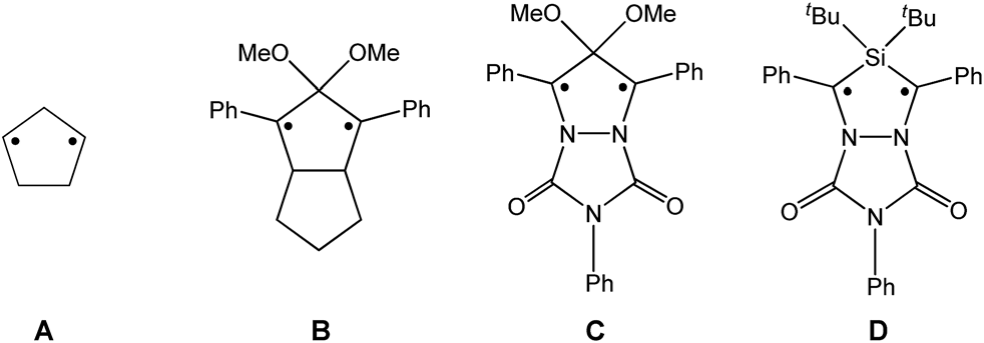
Selected known cyclopentane-1,3-diyl derivatives (**A–D**).

Biradicals and biradicaloids are highly reactive species that can occur in the processes of bond formation and bond breaking. They were discussed as intermediates even in Diels–Alder reactions by M. Dewar *et al.*^1^ Hence, the study of biradicaloids is of general importance. Excellent reviews on this topic were recently published by F. Breher and M. Abe.^2^,^3^ While for cyclopentane-1,3-diyl (Scheme 1, species **A**) several stable main group derivatives are known,^4^–^12^ especially cyclopentane-1,3-diyls are elusive. The parent cyclopentane-1,3-diyl was first observed in 1975 by Buchwalter and Closs, and since then targeted repeatedly by theoretical and *in situ* spectroscopic studies. To date, several heteroatom-substituted derivatives of cyclopentanediyl bearing different substituents are known (selected examples: Scheme 1, species **B–D**).^13^–^26^

**Scheme 2:**
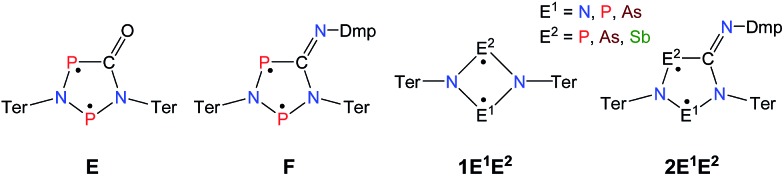
Stable cyclopentane-1,3-diyl derivatives (**E** and **F**), group-15-substituted cyclobutanediyls (**1**) and the targeted cyclopentane-1,3-diyls (**2**).

Until recently, all known cyclobutane-1,3-diyl derivatives incorporated equivalent radical centres, even though several examples investigated by the groups of Power and Yoshifuji are known featuring differing bridging moieties.^27^–^31^ A synthetic protocol was devised by our group, enabling the synthesis of the formal group-15-substituted cyclobutanediyls [As(μ-NTer)_2_P] and [E^1^(μ-NTer)_2_E^2^] (E^1^ = N with E^2^ = P, As, Sb; **1E^1^E^2^** in Scheme 2).^32^,^33^ The reactivity of singlet biradicaloids was mainly studied using the examples of diboradiphosphonio-cyclobutanediyls [^i^Pr_2_P(μ-B^*t*^Bu)]_2_,^35^,^36^ diphosphacyclobutane-diyls [ClC(μ-PMes*)]_2_ (Mes* = 2,4,6-tri-^*tert*^butylphenyl),^37^,^38^ diphosphadiazanediyls [P(μ-NTer)]_2_ (Ter = 2,6-bis(2,4,6-trimethylphenyl)phenyl),^39^,^40^ and digermynes R_2_Ge_2_ (R = 2,6-bis(2,6-diisopropylphenyl)phenyl).^31^,^41^–^43^ For the triazenide-derived species [E^2^(μ-NTer)_2_N], only diminished reactivity was observed, hence these are better regarded as zwitterionic compounds than as biradicaloids in agreement with computational studies. In the case of [Sb(μ-NTer)_2_P], the biradicaloid was found to be a transient intermediate, whose existence could be proven by trapping experiments.^34^

**Scheme 3:**
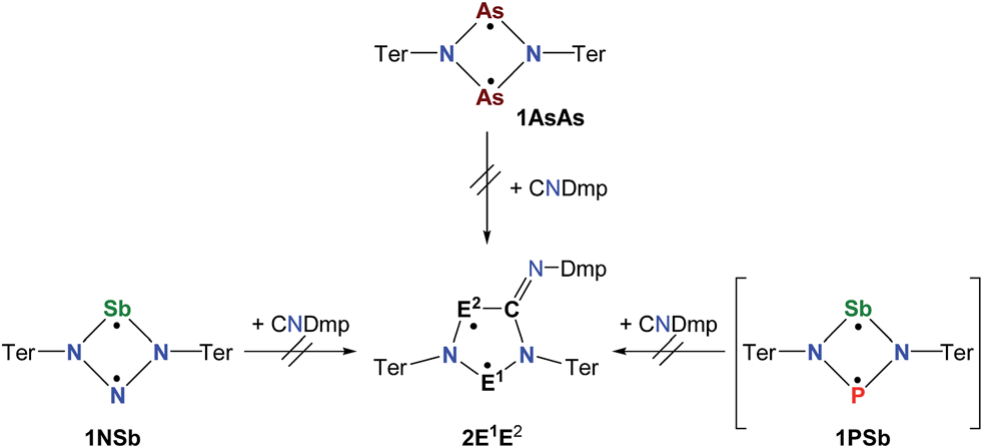
Not accessible cyclopentane-1,3-diyl derivatives (**2AsAs**, **2NSb**, **2PSb**).

**Scheme 4:**
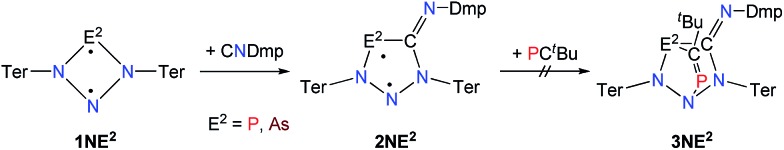
Formation of **2NP** and **2NAs**.

**Scheme 5:**
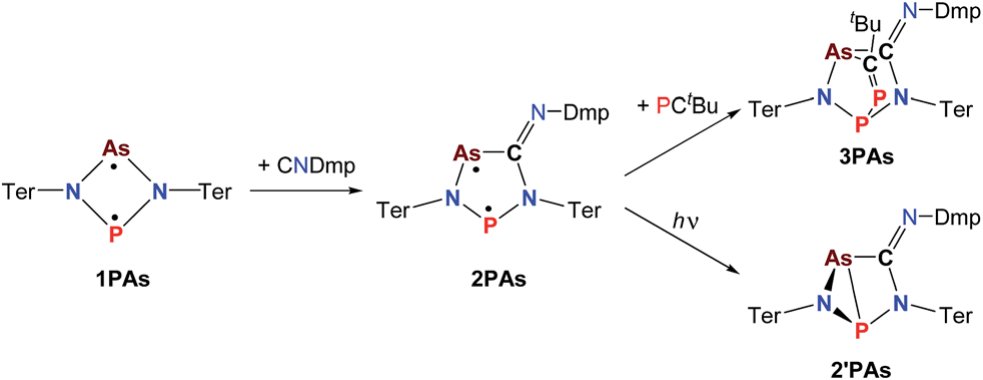
Synthesis and reactivity of P,As-centered cyclopentane-1,3-diyl derivative **2PAs**, housane formation on irradiation (365 nm) and addition reaction to **3PAs**.

A viable access to a stable singlet derivative of formal heteroatomic cyclopentane-1,3-diyls was found in the 1,1-insertion of carbon monoxide, C≡O, into cyclodiphospha diazanediyl, [P(μ-NTer)]_2_ (**1PP**), which afforded species **E** (Scheme 2).^44^ Subsequent systematic investigations targeted the activation of isonitriles, C≡N–R (R = ^*t*^Bu, Dmp, N(SiMe_3_)_2_, Ter; Ter = 2,6-dimesityl-phenyl, Dmp = 2,6-dimethylphenyl), with diphosphadiazanediyl **1PP**. By variation of the organic substituent, steric and electronic properties of the isonitrile could be varied. These could be adjusted to cleanly afford the cyclopentane-1,3-diyl derivative, when 2,6-dimethylphenyl-isonitrile was utilized (species **F**, Scheme 2).^45^ In this contribution, we report on the formation of cyclopentane-1,3-diyls bearing different group 15 radical centres (**2E^1^E^2^**) by reaction of the available group 15 cyclobutanediyl derivatives (**1E^1^E^2^**) with a selected isonitrile, C≡N–Dmp (Schemes 3–5).

## Results and discussion

### Synthesis

Cyclobutanediyl derivatives **1E^1^E^2^** are strongly coloured compounds.^32^,^33^,^46^ Thus, the reactions can often easily be followed visually, apart from reaction monitoring by NMR spectroscopy. While the diphosphadiazanediyl **1PP** readily reacted with 2,6-dimethylphenyl-isonitrile to give **2PP**,^45^ no such transformation was observed when the heavier homologue diarsadiazanediyl **1AsAs** was utilized (Scheme 3). Also, neither the antimony-containing species, stable **1NSb**, nor *in situ* generated **1PSb**, reacted with CNDmp at all.

On the contrary, the ring expansion reaction of the triazenide-derived cyclobutanediyl derivatives **1NP** and **1NAs** proceeded smoothly (Scheme 4) when a solution of CNDmp in benzene was added slowly at ambient temperature to a solution of **1NP** and **1NAs**, respectively, in benzene. Since the starting material **1NP** could not be isolated due to the recurring formation of the triazenide Ter_2_N_3_H as an impurity,^33^ the insertion of the isonitrile and subsequently, the attempted conversion with PC^*t*^Bu, was investigated by means of spectroscopy. The ^31^P NMR spectrum of **1NP** displays a singlet resonance at +342.4 ppm, that shifts to +167.3 ppm upon addition of the isonitrile, indicating the formation of **2NP** in good agreement with 1,2,3,4-triazaphospholes prepared by Müller *et al.* and Jones *et al.* utilizing “click reaction” of azides with phosphaalkynes (*e.g.* C_5_NH_4_–N_3_PC–^*t*^Bu 167.5 ppm).^47^–^50^ It should be noted that various attempts of crystallization only afforded the triazenide Ter_2_N_3_H and the product **2NP** could not be isolated. Upon insertion of the isonitrile, the colour of the solution changed from yellow (**1NP**) to red (**2NP**: *λ*_max_ = 490, calc. 476 nm).^51^,^52^ The attempted addition of PC^*t*^Bu did not alter any of these characteristics, indicating that no reaction with **2NP** occurred in accord with a rather small biradical character (see below).

**Fig. 1:**
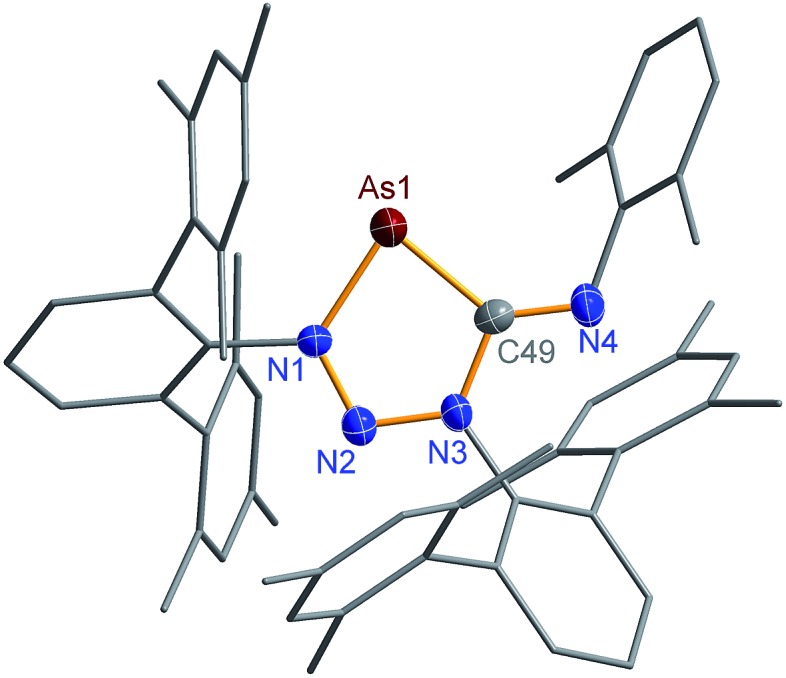
Molecular structure of **2NAs**. Thermal ellipsoids are drawn at 50% probability (173 K). Selected bond lengths [Å] and angles [°]: **2NAs**: As1–N1 1.875(3), As1–C49 1.902(4), N1–N2 1.316(4), N2–N3 1.349(4), N3–C49 1.428(5), N4–C49 1.293(5); N1–As1–C49 82.71(16), N2–N1–As1 119.4(2), N1–N2–N3 109.8(3), N2–N3–C49 119.4(3).

The reaction of **1NAs** with CNDmp similarly resulted in a change of colour from initially yellow (*λ*_max_ = 379 nm) to red, indicating the presence of **2NAs** (*λ*_max_ = 523, calc. 518 nm). Similar to **2NP**, **2NAs** features a *ν*(C=N) vibration at 1612 in the Raman and at 1610 cm^–1^ in the IR spectrum which is significantly different from the *ν*(C≡N) vibration of pure CNDmp exhibiting a CN triple bond (2123 cm^–1^). Crystals suitable for single X-ray studies were obtained after concentration at 4 °C in good yields (83%). Red needle-shaped crystals of **2NAs** decompose above 141 °C and are moisture and air sensitive. Like **2NP**, **2NAs** does not react with PC^*t*^Bu also displaying diminished biradical character. The molecular structure of **2NAs** (Fig. 1) features a planar five-membered N_3_CAs heterocycle. The As–N bond of 1.875(3) Å is considerably longer than in the known tetrazarsole galliumtrichloride adduct Mes*N_4_As·GaCl_3_ (1.784(2), 1.805(2) Å; *cf.* Σ*r*_cov_(As–N) = 1.91 Å) indicating single bond character.^53^ The same holds true for the As–C bond with 1.902(4) Å (Σ*r*_cov_(As–C) = 1.97 Å). The N–N distances in **2NAs** of 1.316(4) and 1.349(4) Å are between the sum of covalent radii for a double and a single bond (1.20, 1.42 Å), indicating delocalized double bond character, while the C49–N3 bond length (1.428(5) Å) corresponds to a single bond (Σ*r*_cov_(C–N) = 1.46 Å) contrary to the exocyclic C49–N4 bond (1.293(5) Å) which is in the typical range of a C=N double bond.^54^

In a next series of experiments we treated a solution of **1PAs** in benzene with CNDmp. Within 10 minutes the insertion of CNDmp into the dark purple **1PAs** (*λ*_max_ = 550 nm)^32^ led in good yields (68%) to a singlet biradicaloid cyclopentanediyl derivative, **2PAs**, which is of green colour (*λ*_max_ = 431, 684, calc. 454, 674 nm, Scheme 5). Interestingly, at the beginning of the reaction the reaction mixture appeared dark grey due to the presence of both the starting material and the reaction product. Astonishingly, the ^31^P NMR shift merely changed from 268.8 to 269.0 ppm. However, as discussed before, upon incorporation of the isonitrile into the four-membered biradicaloid, the *ν*(CN) vibration is dramatically shifted from 2123 to 1633 cm^–1^, as expected for the transition from a C–N triple to double bond. Crystals of **2PAs** decompose above 122 °C.

**Fig. 2:**
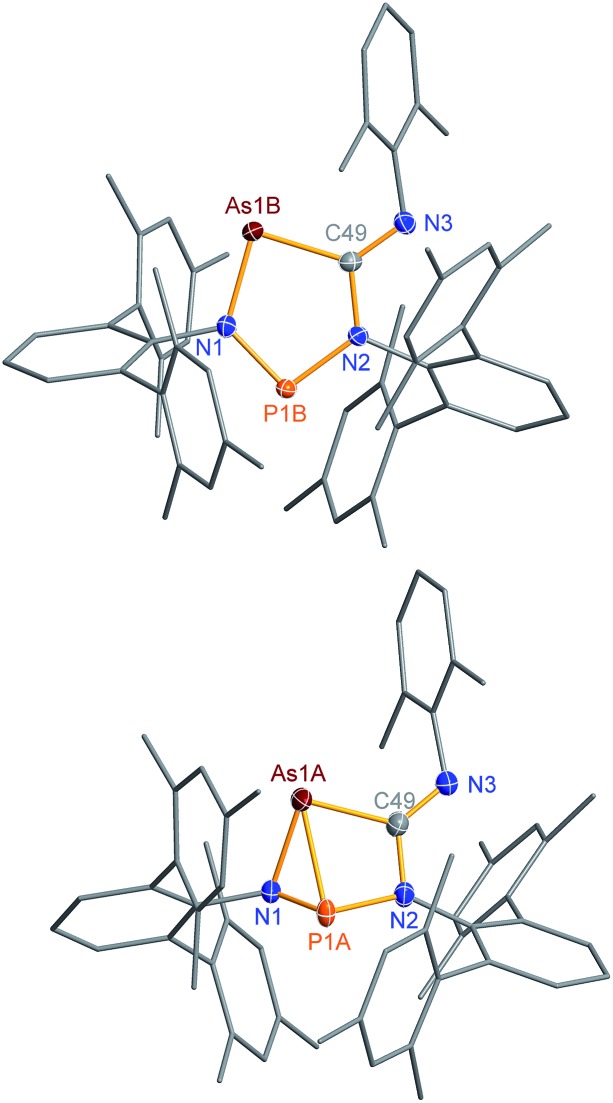
Molecular structure of **2PAs** (top) and **2′PAs** (bottom). Thermal ellipsoids are drawn at 50% probability (123 K). Selected bond lengths [Å] and angles [°]: **2PAs**: As1B–N1 1.874(2), As1B–C49 1.937(2), P1B–N1 1.636(2), P1B–N2 1.691(2), As1B–P1B 3.049(2), P1B–N1–As1B 120.5(1); **2′PAs**: As1A–P1A 2.2920(7), As1A–C49 2.011(2), As1A–N1 1.970(2), P1A–N1 1.692(2), P1A–N2 1.801(2), N1–P1A–N2 95.12(7), P1A–N1–As1A 77.10(6).

The connectivity is furthermore corroborated by the ^13^C NMR data, in which the former isonitrile carbon atom gives rise to a resonance at 184.98 ppm, which appears as doublet with a small *J*_CP_ = 9.9 Hz, indicating a ^2^*J* coupling. X-ray diffraction experiments on single crystals of **2PAs** revealed a secondary irradiation-induced isomerization to the housane isomer **2′PAs** (Scheme 5, Fig. 2, see below), as it was observed for cyclopentanediyl derivatives such as **2PP** indicating substantial biradical character.^44^,^45^ However contrary to **2PP**, for **2PAs** this isomerization did not cause the crystals to completely decompose. Two data sets were collected from a single crystal: the first data set without irradiation prior to measurement and the second after 12 hours of X-ray irradiation on the diffractometer. In both cases, the structural model features disordered P and As atoms. While in the first data set, the planar five-membered species is dominant (86% occupation, Fig. 2 top), in the second data set, which was collected after 12 hours of X-ray irradiation, 95% occupation are found for the housane species (Fig. 2 top). In solution, all attempts to generate **2′PAs** by UV irradiation of **2PAs** led to decomposition, thus no NMR data for **2′PAs** could be obtained.

**Fig. 3:**
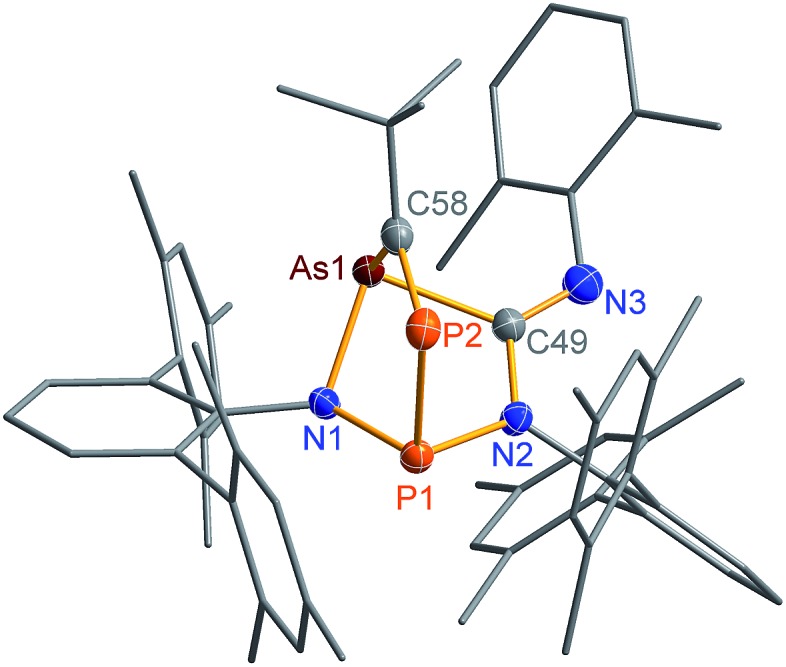
Molecular structure of **3PAs**. Thermal ellipsoids are drawn at 50% probability (173 K). Selected bond lengths [Å] and angles [°]: **3PAs**: As1–N1 1.895(2), As1–C58 1.996(2), As1–C49 2.007(2), P1–N1 1.7378(18), P1–N2 1.756(2), P1–P2 2.2822(8), P2–C58 1.673(2), N3–C49 1.270(3), N1–As1–C58 93.47(9), C58–P2–P1 96.42(8), P1–P2–C58–As1–2.50(13).

The biradical character of **2PAs** invokes high reactivity, which could be demonstrated in the activation of molecules such as phosphaalkynes, PC^*t*^Bu, bearing a P≡C triple bond. The initially green solution of **2PAs** in benzene quickly turned yellow upon addition of the phosphaalkyne and formation of **3PAs** was observed in good yields (78%, Scheme 5, Fig. 3). The ^31^P NMR spectrum exhibited an AB spin system (331.8, 156.8 ppm), indicating that exclusively one isomer was formed. The strong *J*_PP_ coupling of 260 Hz is characteristic for a ^1^*J*_PP_ coupling constant. Single crystal X-ray structure elucidation unequivocally revealed the exclusive formation of one isomer (P–P and C–As bonded species, Fig. 3) therefore featuring complete regioselectivity. This regioselectivity is most probably caused by steric hindrance, as the formation of the putative C–P and P–As bonded isomer would considerably distort the “pocket” formed by the two terphenyls, which is already opened to one side due to insertion of the isonitrile (difference between both isomers: Δ*E* = 48 kJ mol^–1^). It is interesting to note that for the cyclobutanediyl derivative [As(μ-NTer)_2_P], the regioselectivity was opposite and only the C–P and P–As bonded isomer was observed due to thermodynamic preference of a C–P over C–As bond.^32^

Single crystals of **2PAs**/**2′PAs** and **3PAs** suitable for structure analysis were obtained from benzene solutions. The most prominent structural feature of **2PAs** represents the almost planar five-membered heterocycle with rather short P–N bond lengths (P1B–N1 1.636(2), P1B–N2 1.691(2) Å) in the range of PN double bonds (Σ*r*_cov_(P=N) = 1.62 Å), while the As–N (As1B–N1 1.874(2) Å) and As–C bonds (As1B–C49 1.937(2) Å, Fig. 2) are in the range of single bonds (see also **2NAs**, Fig. 1).^53^,^55^,^56^

The structure changes dramatically upon irradiation and formation of the housane **2′PAs**. The transannular P–As distance is shortened from 3.049(2) to 2.2920(7) Å clearly indicating the presence of a transannular P–As single bond (Σ*r*_cov_(P–As) = 2.32 Å). Additionally, the P–N–As angle strongly decreases from 120.5(1) to 77.10(6)°. The three-membered As–N–P ring is almost perpendicular condensed to the four-membered As–P–N–C ring. These experimental structural parameters are in good agreement with those of DFT computations (see below and ESI†).

The phosphaalkyne addition product **3PAs** shows a puckered five-membered ring with a transannular P–As distance of 2.918(2) Å. The P–C bridging bond length amounts to 1.673(2) Å in accord with a P=C double bond.

### Computations – bonding and biradical character

**Fig. 4:**
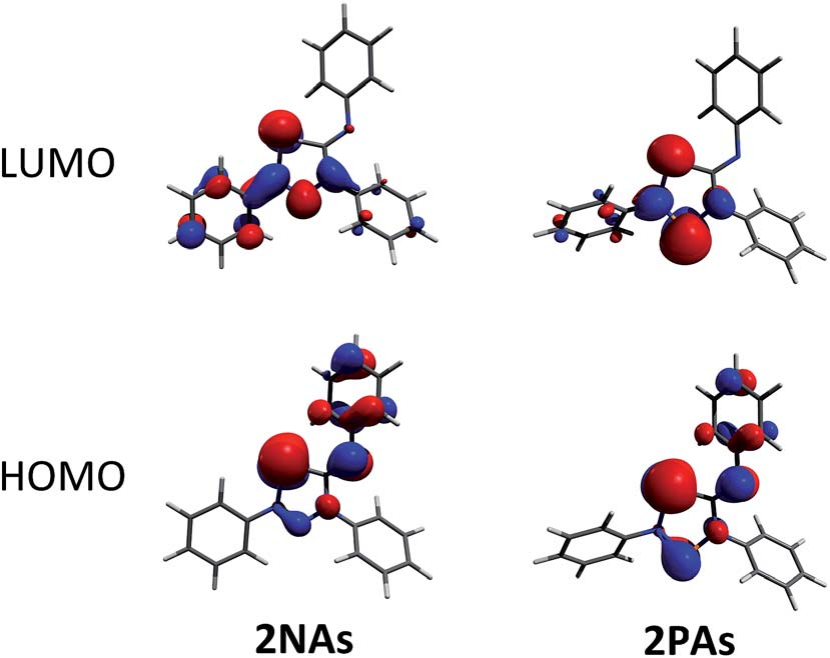
Frontier orbitals of **2NAs** (left) and **2PAs** (right). For the orbital representations phenyl-substituted model compounds are used for clarity.

**Scheme 6:**
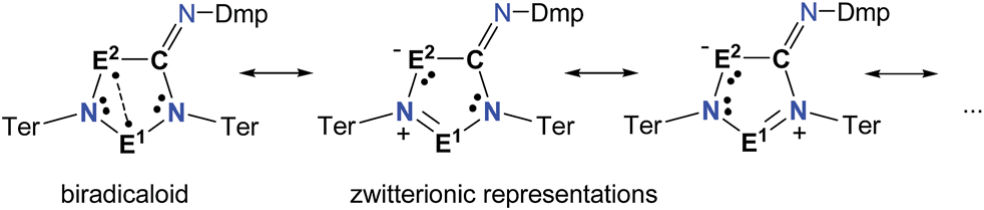
Selected Lewis representations according to NBO analysis.

To shed some light into the bonding and biradical character, MO (Fig. 4), NBO (Scheme 6) and CASSCF computations have been carried out. MO and NBO computations show formal 6π electronic **2E^1^E^2^** five-membered heterocycles (Table 1). A common electronic feature of the heterocycles **2NP**, **2NAs**, and **2PAs** is the weak aromaticity as indicated by NICS values (Table 1).^57^ The frontier orbitals feature a p-type transannular antibonding π-HOMO and transannular bonding π*-LUMO between the radical centres, in accord with other group 15 singlet biradicaloids (Fig. 4).

**Table 1:** Computational data for the cyclopentanediyls **2E^1^E^2^** with Ter substituents

	2NP	2NAs	2NSb^*a*^	2 PAs	2PSb^*a*^	2AsAs^*a*^
*λ*_max_calc.	476	518	542	674	715	750
Δ*E*_form_^*b*^	–148.0	–114.6	–55.6	–94.8	–51.4	–91.2
S–T gap^*b*^	–148.9	–132.7	–118.8	–75.4	–72.7	–44.4
*β* ^*c*^	13%	11%	4%	24%	7%	38%
*c*_1_	0.946	0.956	0.961	0.916	0.982	0.889
*c*_2_	–0.250	–0.229	–0.132	–0.339	–0.185	–0.433
BO(E–E)^*f*^	0.313	0.288	0.262	0.389	0.343	0.434
π e cpd^*d*^	6.51	6.51	6.55	6.49	6.53	6.50
NICS(0)^*e*^	–8.32	–7.45	–6.40	–5.62	–4.84	–4.66
NICS(1)^*e*^	–6.65	–6.10	–5.51	–3.24	–3.18	–2.90

^*a*^Species not isolated.

^*b*^Calculated in [kJ mol^–1^]: Δ*E*_form_ = *E*(**2**) – [*E*(**1**) + *E*(CNDmp)].

^*c*^ *β* = 2*c*_2_^2^/(*c*_1_^2^ + *c*_2_^2^), the two main contributions according to CASSCF(6,6) computations were used.

^*d*^π electrons in cpd = cyclopentanediyl, occupation of p_*z*_ orbitals in the five-membered heterocycle according to NBO analysis.

^*e*^In ppm.

^*f*^Wiberg bond index.

To investigate the biradicaloid character of **2E^1^E^2^**, the singlet–triplet energy gap (Δ*E*_S–T_) was computed for **2E^1^E^2^** and CASSCF(6,6) computations were carried out (CASSCF = complete active space self-consistent field). Experimentally, biradicaloids **2E^1^E^2^** show no EPR signal and ^1^H, ^13^C, and ^31^P NMR signals. All **2E^1^E^2^** compounds have a singlet ground state in accord with rather large Δ*E*_S–T_ values (Table 1) significantly decreasing the heavier the group 15 elements E^1^ and E^2^. CASSCF(6,6) computations confirmed the biradicaloid nature of **2E^1^E^2^**. The dominant contributions to the CI wave function arise from the HOMO/LUMO exchange. The biradicaloid character can be estimated by using the formula: *β* = 2*c*_2_^2^/(*c*_1_^2^ + *c*_2_^2^).^58^ Hence, upon insertion of the isonitrile into the four-membered ring of **1** the biradical character is preserved compared to the starting material **1E^1^E^2^**. Therefore, as illustrated in Scheme 6 and Table 1, the zwitterionic character increases (biradical character decrease) along E^1^ = As < P < N and E^2^ = Sb < As < P. For example, a biradical character *β* of only 13% was computed for **2NP** and 11% for **2NAs**, respectively (CASSCF(6,6), coefficients of main contributions 0.946, –0.250 for **1NP** and 0.956, –0.229 for **1NAs**).^58^ However, **2PAs** features substantial biradical character of *β* = 24% (CASSCF(6,6), *c*_1_ = 0.916 and *c*_2_ = –0.339), in agreement with the experimental fact that this species is capable of activating molecules containing triple bonds (*vide supra*) contrary to **2NP** or **2NAs**. Moreover, the larger zwitterionic character of **2NAs** compared to **2PAs** is also manifested by the HOMO of **2NAs** featuring very large coefficients at As and very small ones at N, while for **2PAs** the contributions are distributed almost evenly.

The computational data show a correlation between biradical character *β* and Wiberg bond index (WBI) between the two radical centers (Table 1). The WBI(E^1^–E^2^) of all considered species **2E^1^E^2^** ranges from 0.262 to 0.434, which originates from partial occupation of the transannularly bonding LUMO. This reflects strong antiferromagnetic coupling between the radical centers E^1^ and E^2^. NICS values (between –2.9 and –8.3, Table 1, *cf.* benzene –11.5 ppm, azulene –21.5 [5 ring] and –8.3 [7 ring] ppm) decrease when heavier elements are incorporated in the five-membered ring, indicating less stabilization by electron delocalization within the heterocycle, which is due to diminished orbital overlap between E and the adjacent N or C atoms.

**Scheme 7:**
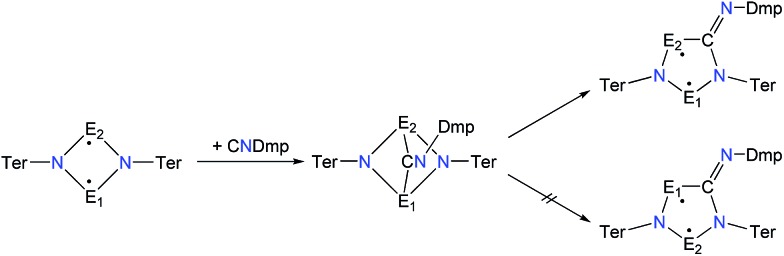
Proposed reaction mechanism for isonitrile activation with **1E^1^E^2^** (E^1^ is lighter than E^2^).

In analogy to the activation of CO with **1PP** and the reaction of **1PP** with different isonitriles, we suggest a mechanism involving the formation of a [1.1.1]bicyclic intermediate, which subsequently rearranges to give the cyclopentanediyl derivative (Scheme 7).^44^,^45^ The formation of the [1.1.1]bicyclic species is endothermic for all species **2E^1^E^2^** with E^1^ or E^2^ being N, increasing in the order As (62.8) < P (82.0) < Sb (126.8 kJ mol^–1^) for E. For the heavier homologues with E^1^ being P, it is exothermic and the reaction energy increases in the same order: E^2^ = As (–68.8) < P (–49.9) < Sb (–28.9 kJ mol^–1^). The second reaction step, the rearrangement from the [1.1.1]bicycle to the planar five-membered ring, is exothermic in every case. In this case, there is a tendency of the reaction becoming less exothermic as the pnictogen E^2^ becomes heavier (Δ_R_E: N > P > As > Sb; *e.g.***2PAs** –66.5, **2PAs** –30.9, **2PSb** –7.0; Table S3†), with the exception of E^1^ = N and E^2^ = Sb, which is slightly more exothermic than for E^2^ = As. In all combinations of E^1^ and E^2^ being N, P, As, or Sb, the overall insertion reaction is exothermic (*e.g.***2NP** –148.0, **2NAs** –114.6, **2NSb** –55.6 kJ mol^–1^; Tables S2 and S3†). Interestingly, all cyclopentanediyl derivatives **1** in which E^1^ is heavier than E^2^ are thermodynamically more stable than the observed species, in which E^2^ is heavier than E^1^. This means, that the observed products are formed owing to kinetic reaction control. This is plausible, since the heavier E–N bonds are weaker and hence more readily activated. This can be corroborated with computed transition states for the rearrangement from [1.1.1]bicycle to planar five-membered ring for the example of a phenyl-substituted model compound of **2PAs**, in which the insertion into the N–As bond requires 16 kJ mol^–1^ less activation energy than into the N–P bond.

Finally, we want to address the issue of housane formation. Computational studies indicate, that **2PAs** is more favorable than the housane isomer **2′PAs** by 94 kJ mol^–1^. The computed activation barrier for the formation of the P–As bond amounts to 167 kJ mol^–1^ and breaking the bond 73 kJ mol^–1^. These values are higher than computed for the previously investigated housanes (42 kJ mol^–1^ difference in energy, activation barrier of 83 kJ mol^–1^).^45^ This provides an explanation for the slower decomposition in the X-ray beam of the diffractometer, which allowed the structure determination of **2PAs** as well as **2′PAs**. However, upon UV irradiation, decomposition occurred, preventing the isolation of the housane species **2′PAs**.

**Fig. 5:**
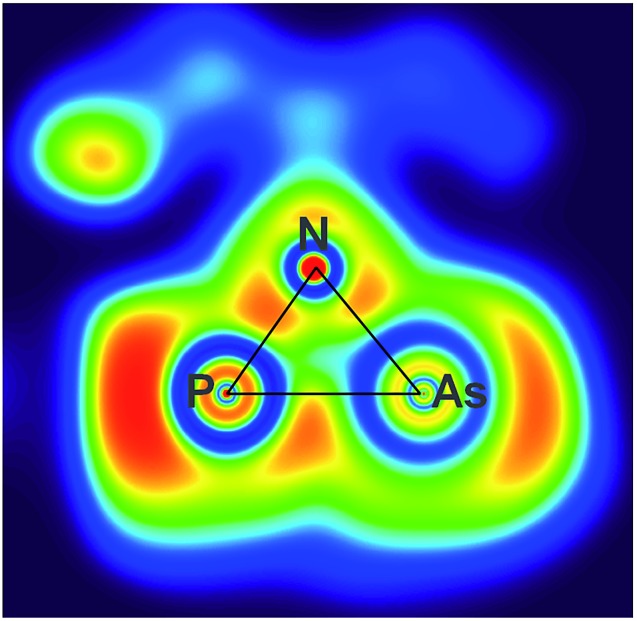
ELF representation of **2P′As** utilizing a phenyl substituted model compound for clarity. A section of the N–P–As plane is shown. The maximum is located aside the axis between As (left) and P (right).

The electronic situation of both isomers clearly differs, as the housane features a bent bond between the former radical centres, while in the biradicaloid there is no direct interaction between P and As. This is apparent from the maximum in the ELF (electron localization function) aside the P–As axis of **2′PAs**, which also features a localized double bond (Fig. 5).

## Conclusions

In summary, the ring expansion reaction of cyclobutanediyls with isonitriles enabled the synthesis of three new group 15 derivatives of cyclopentane-1,3-diyl featuring N/P, N/As, or P/As atoms as radical centres within the five-membered heterocycle. However, there are limitations to this insertion reaction, and the preparation of cyclopentane-1,3-diyls bearing N/Sb, As/As, or P/Sb radical centres remains a challenge. Two reasons can be accountable for this: (i) in the zwitterionic border case, due to strong polarization the valence electron density distributed far from equally between the formal radical centres, so the biradical reactivity is diminished, and (ii) by incorporating heavier elements in the heterocycle (*e.g.* within the homologous series **2NP**, **2NAs**, **2NSb**), the distance between the radical centres is large and hence the orbital overlap is small, thereby reducing the stability of the heavier cyclopentane-1,3-diyl species. This is reflected in the decreasing relative stability of the singlet ground state compared to the lowest lying triplet state.

The new cyclopentane-1,3-diyl derivatives containing an N_3_ moiety (E^1^ = N) have strongly polarized N–E^2^ bonds, a rather small biradical character and therefore are better referred to as zwitterions, which is also manifested by their inability to activate molecules bearing multiple bonds. In contrast, the P/As centered biradicaloid **2PAs** exhibits a considerable biradical character, higher reactivity and can be isomerized to the short-bond species **2′PAs** or be utilized in small molecule activation.

## Supplementary Material

Supplementary information

Crystal structure data
