# Supplementary material for: Zwitterionic and biradicaloid heteroatomic cyclopentane derivatives containing different group 15 elements
Source: Chem Sci. 2015 Oct 27;7(1):745–51. doi: 10.1039/c5sc03515e (PMC5953028; doi:10.1039/c5sc03515e)
Supplement: Supplementary file 1 [file SC-007-C5SC03515E-s001.pdf]

Electronic Supplementary Material (ESI) for Chemical Science.
This journal is © The Royal Society of Chemistry 2015

Supporting Information

Zwitterionic And Biradicaloid Heteroatomic Cyclopentane Derivatives Containing Different Group 15 Elements

Alexander Hinz, Axel Schulz, and Alexander Villinger*

This file includes:

1. General Information 2
2. Syntheses 3
   2.1. Synthesis of $Ter_2N_3AsCNDmp$ (**2NAs**) 3
   2.2. Synthesis of $Ter_2N_2PAsCNDmp$ (**2PAs**) 4
   2.3. Synthesis of $Ter_2N_2PAsCNDmpPC^tBu$ (**3PAs**) 5
   2.4. Attempted synthesis of $Ter_2N_3PCNDmp$ (**2NP**) 6
3. Crystallographic Data 7
   3.1. Numbering scheme of **2NAs** 8
   3.2. Numbering scheme of **2PAs** 9
   3.1. Numbering scheme of **2'PAs** 10
   3.2. Numbering scheme of **3PAs** 11
4. Computational Details 12
   4.1. Optimized geometries 13
      4.1.1. $Ter_2N_3PCNDmp$ 13
      4.1.2. $Ter_2N_3AsCNDmp$ 14
      4.1.3. $Ter_2N_3SbCNDmp$ 16
      4.1.4. $Ter_2N_2PAsCNDmp$ 18
      4.1.5. $Ter_2N_3PSbCNDmp$ 20
      4.1.6. $Ter_2N_2As_2CNDmp$ 22
      4.1.7. $Ter_2N_3PAsCNDmpPC^tBu$ 23
      4.1.8. $Ter_2N_3PAsCNDmpPC^tBu$ – isomeric species 25
      4.1.9. CNDmp 27
   4.2. Analysis of Cyclopentane-1,3-diyl derivatives 29
5. References 32

1. General Information

All manipulations were carried out under oxygen- and moisture free conditions using standard Schlenk and Drybox techniques. [P(μ-NTer)]$_2$,[1] [As(μ-NTer)]$_2$,[2] [E(μ-NTer)$_2$N] (E = P, As, Sb),[3] [Sb(μ-NTer)$_2$P],[4] and CNDmp[5] were prepared according to literature procedures. Fluorobenzene was dried over CaH_2, distilled and degassed prior to use. Diethyl ether, THF, toluene and benzene were dried over Na/benzophenone and freshly distilled prior to use.

NMR: $^{31}P\{^1H\}$, $^{13}C\{^1H\}$ and ^1H NMR spectra were recorded on BRUKER spectrometers AVANCE 250, AVANCE 300 and AVANCE 500, respectively. The ^1H and ^{13}C NMR chemical shifts were referenced to the solvent signals.[6] The ^{31}P NMR chemical shifts are referred to H_3PO_4 (85%) respectively. C_6D_6 was dried over Na and freshly distilled prior to use.

CHN analysis: Analysator Flash EA 1112 from Thermo Quest.

IR: Nicolet 380 FT-IR with a Smart Orbit ATR module.

RAMAN: LabRAM HR 800 Horiba Jobin YVON equipped with a High Stability BX40 Microscope (Focus 1 µm) or an Olympus Mplan 50xNA 0.70 lens, the laser is variable and was chosen prior to the measurement.

DSC: DSC 823e from Mettler-Toledo (Heating rate 5 °C/min).

MS: Finnigan MAT 95-XP from Thermo Electron was used.

X-ray Structure Determination: X-ray quality crystals of all compounds were selected in Fomblin YR-1800 perfluoroether (Alfa Aesar) at ambient temperatures. The samples were cooled to 173(2) K during measurement. The data were collected on a Bruker Apex Kappa-II CCD diffractometer or on a Bruker-Nonius Apex X8 CCD diffractometer using graphite monochromated Mo K_α radiation (λ = 0.71073). The structures were solved by direct methods (*SHELXS-2013*)[7] and refined by full-matrix least squares procedures (*SHELXL-2013*).[8] Semi-empirical absorption corrections were applied (SADABS).[9] All non-hydrogen atoms were refined anisotropically, hydrogen atoms were included in the refinement at calculated positions using a riding model.

2. Syntheses

2.1. Synthesis of $Ter_2N_3AsCNDmp$ (2NAs)

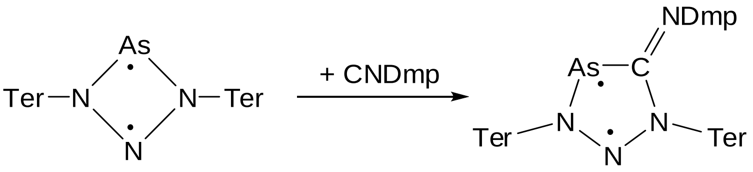

To a solution of $[As(\mu\text{-}NTer)_2N]$ (165 mg, 0.222 mmol) in benzene (4 ml), a solution CNDmp (30 mg, 0.229 mmol) in benzene (2 ml) was added dropwise at ambient temperature. Immediately, a change of colour from yellow to red occurred. After the addition, the solution was concentrated to approx. 1 ml and left undisturbed at 4 °C, affording red needle-shaped crystals. The supernatant was removed via syringe and the crystals were dried in vacuo (161 mg, 0.184 mmol, 83%).

Mp. 141 °C (dec.). **EA** for $C_{63}H_{65}AsN_4$ found (calc.) C 79.11 (79.39), H 6.67 (6.87), N 5.74 (5.88). **^{1}H NMR** (298 K, C_6D_6, 250.1 MHz): 1.66 (s, 6 H, CH_3), 1.72 (s, 6 H, CH_3), 1.85 (s, 12 H, CH_3), 2.15 (s, 6 H, CH_3), 2.21 (s, 6 H, CH_3), 2.27 (s, 6 H, CH_3), 6.88 (s, 4 H, CH_{Mes}), 6.72 (s, 4 H, CH_{Mes}), 6.76-7.02 (m, 9 H). **^{13}C{^{1}H} NMR** (298 K, C_6D_6, 62.9 MHz): 19.26 (s, CH$_3$), 21.23 (s, CH$_3$), 21.42 (s, CH$_3$), 21.52 (s, CH$_3$), 21.66 (s, CH$_3$), 21.96 (s, CH$_3$), 122.78 (s, *C*H), 128.92 (s, *C*H), 129.09 (s, *C*H), 129.21 (s, *C*H), 129.25 (s, *C*H), 129.45 (s, *C*H), 129.86 (s, *C*H), 132.53 (s, *C*H), 132.65 (s, *C*H), 136.31 (s), 136.56 (s), 136.81 (s), 136.94 (s), 137.11 (s), 137.42 (s), 137.84 (s), 137.94 (s), 138.10 (s), 138.34 (s), 141.10 (s), 142.09 (s), 152.43 (s), 181.05 (s, As*C*N). **UV/vis** (λ_{max}, nm): 350 (br), 523. **IR** (ATR, cm^{-1}): 536 (m), 543 (m), 551 (m), 559 (m), 574 (m), 586 (m), 594 (m), 613 (s), 638 (m), 655 (m), 676 (m), 730 (s), 742 (m), 759 (vs), 767 (s), 786 (m), 808 (m), 848 (s), 879 (w), 908 (w), 919 (w), 966 (m), 1031 (m), 1054 (m), 1095 (m), 1157 (s), 1201 (s), 1222 (s), 1257 (m), 1274 (m), 1295 (s), 1375 (m), 1411 (m), 1450 (s), 1481 (m), 1535 (vs), 1556 (m), 1594 (w), 1610 (w), 2730 (w), 2854 (w), 2914 (m), 2944 (m), 2998 (w). **Raman** (632 nm, cm^{-1}): 231 (43), 246 (51), 264 (36), 279 (27), 331 (23), 368 (5), 386 (17), 404 (13), 423 (23), 435 (12), 468 (9), 478 (13), 492 (11), 499 (12), 512 (15), 521 (39), 552 (43), 559 (55), 575 (100), 613 (16), 638 (13), 654 (15), 700 (11), 742 (12), 756 (11), 765 (7), 774 (7), 804 (6), 833 (7), 855 (19), 911 (4), 944 (11), 991 (7), 1004 (19), 1054 (9), 1076 (12), 1083 (11), 1162 (21), 1188 (8), 1201 (16), 1222 (6), 1258 (8), 1286 (37), 1304 (59), 1380 (14), 1414 (10), 1456 (36), 1481 (18), 1523 (9), 1535 (9), 1574 (13), 1585 (15), 1612 (21), 2730 (1), 2854 (1), 2918 (4), 2949 (2), 3008 (1). **MS** (CI, pos., isobutane) m/z (%): 132 (49) $[CNDmp]^+$, 716 (13), 743 (100) $[Ter_2N_3As]^+$, 874 (<1) $[M]^+$.

2.2. Synthesis of $Ter_2N_2PAsCNDmp$ (2PAs)

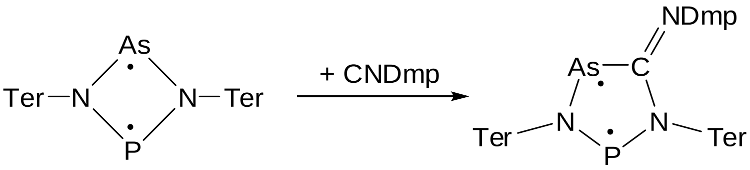

To a violet solution of [P(μ-NTer)$_2$As] (181 mg, 0.246 mmol) in 4 ml benzene, a colourless solution of CNDmp (33 mg, 0.252 mmol) in 2 ml was added within 5 minutes. The solution turned grey and finally green within 10 minutes. Afterwards, volatiles were removed in vacuo until crystallization commenced (approx. 0.5 ml). The solution was left undisturbed overnight, resulting in the deposition of a green precipitate. The supernatant was filtered off (sintered glass frit) and the solid was dried in vacuo (150 mg, 0.168 mmol, 68%).

Mp. 122 °C (dec.). **EA** for $C_{57}H_{59}N_3AsP$ found (calc.): C 76.20 (76.75), H 6.93 (6.67), N 4.38 (4.71). **^{1}H NMR** (298 K, C_6D_6, 300 MHz): 1.71 (s, 6 H, CH_3), 1.72 (s, 6 H, CH_3), 1.95 (s, 12 H, CH_3), 2.08 (s, 6 H, CH_3), 2.25 (s, 6 H, CH_3), 2.28 (s, 6 H, CH_3), 2.30 (s, 6 H, CH_3), 6.58 (s, 1 H, CH_{Mes}), 6.61 (s, 1 H, CH_{Mes}), 6.70-6.96 (m, 12 H, CH). **^{13}C{^{1}H} NMR** (298 K, C_6D_6, 62.9 MHz): 19.00 (s, CH$_3$), 19.08 (s, CH$_3$), 20.64 (s, CH$_3$), 21.37 (s, CH$_3$), 21.50 (s, CH$_3$), 21.66 (s, CH$_3$), 21.71 (s, CH$_3$), 21.74 (s, CH$_3$), 21.80 (s, CH$_3$), 21.93 (s, CH$_3$), 122.59 (s, CH), 128.21 (s, CH), 128.24 (s, CH), 129.08 (s, CH), 129.18 (s, CH), 129.26 (s, CH), 129.40 (s, CH), 129.45 (s, CH), 129.53 (s, CH), 130.20 (s, CH), 131.71 (s, CH), 132.61 (s, CH), 135.24 (s), 135.43 (d, J_{CP} = 12.7 Hz), 136.39 (s), 136.80 (s), 137.18 (s), 137.39 (s), 137.56 (s), 137.88 (s), 138.36 (s), 139.92 (d, J_{CP} = 12.1 Hz), 141.20 (d, J_{CP} = 5.0 Hz), 151.43 (s), 184.98 (d, J_{CP} = 9.9 Hz). **^{31}P NMR** (298 K, C_6D_6, 121.5 MHz): 269.0 (s). **Raman** (632 nm): 3067 (5), 3044 (7), 3018 (7), 2919 (30), 2858 (8), 2731 (3), 1681 (8), 1633 (88), 1616 (34), 1594 (100), 1584 (48), 1467 (16), 1422 (15), 1406 (17), 1382 (17), 1307 (55), 1269 (16), 1256 (16), 1222 (7), 1178 (43), 1118 (2), 109 (17), 1087 (35), 1073 (36), 1010 (7), 947 (8), 903 (2), 874 (21), 852 (6), 843 (11), 812 (6), 794 (6), 774 (7), 767 (3), 744 (9), 691 (4), 660 (1), 643 (8), 616 (35), 598 (3), 579 (81), 564 (37), 557 (34), 529 (22), 508 (21), 487 (4), 474 (7), 448 (16), 415 (20), 400 (22), 390 (12), 380 (5), 360 (38), 345 (14), 310 (5), 262 (25), 237 (28), 217 (7). **IR** (ATR, cm^{-1}): 2967 (w), 2943 (w), 2912 (m), 2850 (m), 2727 (w), 1630 (w), 1610 (m), 1574 (w), 1531 (vs), 1443 (s), 1406 (s), 1371 (m), 1335(vw), 1309 (vw), 1286 (w), 1273 (vw), 1240 (w), 1221 (m), 1192 (s), 1163 (w), 1138 (w), 1097 (m), 1088 (m), 1080 (m), 1024 (s), 990 (w), 957 (vw), 945 (vw), 937 (vw), 908 (w), 867 (w), 847 (vs), 827 (w), 806 (w), 798 (s), 773 (w), 762 (s), 746 (s), 698 (w), 690 (w), 673 (m), 654 (vw), 644 (w), 600 (m), 573 (m), 559 (w), 546 (vw), 528 (vw). **MS** (CI, pos., isobutane) m/z (%): 132 (57) [CNDmp]$^+$, 188 (10), 330 (100) [TerNH$_3$]$^+$, 372 (11), 386 (24) [TerNH$_2$+C$_4$H$_9$]$^+$, 459 (35) [TerNCNDmp]$^+$, 687 (71) [Ter$_2$N$_2$PH$_2$]$^+$, 705 31) [Ter$_2$N$_2$PAs]$^+$, 743 (14), 760 (11) [Ter$_2$N$_2$PAs]$^+$, 793 (26), 829 (9).

2.3. Synthesis of $Ter_2N_2PAsCNDmpPC^t Bu$ (3PAs)

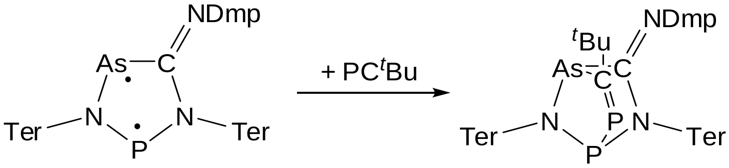

To a solution of [Ter_2N_2PAs(CNDmp)] (121 mg, 0.134 mmol) in benzene (3 ml), PC^{t}Bu (20 µl) was added via microliter syringe. After shaking of the reaction mixture, the solution turned yellow within 30 minutes. The yellow solution was concentrated until crystallization commenced and left undisturbed at ambient temperature overnight, affording yellow crystals. The supernatant was removed via syringe and the crystals were dried in vacuo (93 mg, 0.094 mmol, 70%).

Mp. 118 °C (dec.). **EA** for $C_{62}H_{68}AsN_3P_2$ found (calc.): C 75.20 (75.06), H 6.93 (6.91), N 4.38 (4.24). **^{1}H NMR** (298 K, C_6D_6, 300 MHz): 1.00 (s, 9 H, ^{t}Bu), 1.72 (s, 3 H, CH_3), 2.02 (s, 3 H, CH_3), 2.06 (s, 9 H, CH_3), 2.11 (s, 3 H, CH_3), 2.21 (s, 3 H, CH_3), 2.28 (s, 3 H, CH_3), 2.30 (s, 3 H, CH_3), 2.34 (s, 3 H, CH_3), 2.36 (s, 3 H, CH_3), 2.37 (s, 3 H, CH_3), 2.38 (s, 3 H, CH_3), 2.47 (s, 3 H, CH_3), 6.45 (dd, J_{HH} = 2.1, J_{HH} = 7.2 Hz, 1 H, C*H*), 6.58 (s, 1 H, C*H*), 6.61 (s, 1 H, C*H*), 6.65 (dd, J_{HH} = 1.7, J_{HH} = 7.5 Hz, 1 H, C*H*), 6.70-6.73 (m, 3H), 6.79-6.94 (m, 9 H), 6.97 (t, J_{HH} = 7.4, 1 H, C*H*). **^{13}C{^{1}H} NMR** (298 K, C_6D_6, 62.9 MHz): 17.88 (s, *C*H_3), 20.64 (s, *C*H_3), 20.68 (s, *C*H_3), 20.79 (s, *C*H_3), 21.09 (s, *C*H_3), 21.53 (s, *C*H_3), 21.67 (s, *C*H_3), 21.68 (s, *C*H_3), 22.37 (d, J_{CP} = 6.4 Hz, *C*H_3), 21.45 (d, J_{CP} = 12.7 Hz, *C*H_3), 22.49 (s, *C*H_3), 22.68 (s, *C*H_3), 22.80 (s, *C*H_3), 23.02 (s, *C*H_3), 23.05 (s, *C*H_3), 24.39 (d, J_{CP} = 7.5 Hz, *C*H_3), 33.49 (d, J_{CP} = 11.8 Hz, C(*C*H_3)$_3$), 41.69 (d, J_{CP} = 9.7 Hz, *C*(CH_3)$_3$), 122.68 (s, *C*H), 123.14 (s, *C*H), 127.18 (s, *C*H), 127.54 (s, *C*H), 127.80 (s, *C*H), 128.30 (s, *C*H), 128.49 (s, *C*H), 128.68 (s, *C*H), 128.92 (s, *C*H), 129.11 (s, *C*H), 129.18 (s, *C*H), 129.26 (s, *C*H), 129.41 (s, *C*H), 129.54 (s, *C*H), 130.22 (s, *C*H), 130.71 (s, *C*H), 131.68 (s, *C*H), 131.98 (s, *C*H), 132.63 (s, *C*H), 133.18 (s, *C*H), 133.23 (d, J_{CP} = 10.1 Hz), 135.45 (s), 135.72 (d, J_{CP} = 3.2 Hz), 136.02 (s), 136.15 (s), 136.26 (s), 137.14 (s), 137.19 (s), 137.26 (s), 137.37 (s), 137.42 (s), 137.46 (s), 137.57 (s), 137.64 (s), 137.85 (s), 137.93 (d, J_{CP} = 14.0 Hz), 137.98 (s), 138.05 (s), 138.15 (s), 138.78 (d, J_{CP} = 5.3 Hz), 138.79 (s), 139.05 (d, J_{CP} = 1.3 Hz), 129.27 (d, J_{CP} = 2.1 Hz), 139.55 (s), 139.60 (s), 140.29 (s), 141.05 (d, J_{CP} = 11.8 Hz), 142.37 (d, J_{CP} = 3.2 Hz), 143.28 (d, J_{CP} = 7.5 Hz), 149.03 (s), 174.31 (d, J_{CP} = 9.6 Hz), 220.03 (d, J_{CP} = 81.7 Hz). **^{31}P NMR** (298 K, C_6D_6, 121.5 MHz): 331.8 ($^2J_{PP}$ = 260 Hz, *P*=C), 156.8 ($^2J_{PP}$ = 260 Hz, N*P*N). **IR** (ATR, cm^{-1}): 2956 (m), 2916 (m), 2854 (w), 2729 (vw), 1608 (s), 1574 (s), 1444 (s), 1398 (s), 1373 (m), 1360 (w), 1259 (s), 1232 (m), 1196 (s), 1165 (vw), 1092 (s), 1070 (sh), 1026 (s), 976 (m), 881 (w), 847 (s), 795 (vs), 764 (w), 750 (m), 675 (w), 640 (vw), 604 (vw), 594 (vw), 577 (vw), 567 (vw), 552 (vw). **Raman** (632 nm, cm^{-1}): 3065 (14), 3042 (26), 3008 (20), 2951 (27), 2915 (75), 2854 (21), 2767 (2), 2724 (6), 2700 (2), 1612 (36), 1578 (39), 1481 (16), 1462 (13), 1437 (20), 1417 (11), 1399 (28), 1379 (26), 1373 (23), 1303 (100), 1282 (27), 1258 (12), 1251 (14), 1227 (29), 1211 (20), 1198 (26), 1188 (20), 1160 (15), 1102 (38), 1096 (34), 1071 (29), 1025 (7), 1005 (16), 995 (31), 985 (5), 946 (9), 926 (5), 922 (5), 881 (3), 848 (5), 834 (5), 819 (3), 808 (1), 797 (3), 789 (4), 771 (7), 742 (29), 682 (10), 655 (5), 605 (3), 595 (16), 580 (71), 552 (51), 539 (12), 526 (53), 515 (24), 489 (17), 432 (29), 418 (61), 399 (21), 387 (10), 375 (7), 357 (22), 335 (23), 314 (3), 281 (9), 270 (12), 263 (11), 234 (32), 210 (13). **MS** (CI, pos., isobutane) m/z (%): 132 (40) [CNDmp]$^+$, 330 (52) [TerNH_3]$^+$, 358 (19) [TerNP]$^+$, 459 (69) [TerNCNDmp+H]$^+$, 533 (84) [M–TerNCNDmp]$^+$, 687 (32) [(TerNH)$_2$P]$^+$, 705 (27), 760 (10) [Ter_2N_2PAs]$^+$, 860 (9) [M–CNDmp]$^+$, 891 (3) [M–PC^{t}Bu]$^+$, 992 (26) [M+H]$^+$.

2.4. Attempted synthesis of $Ter_2N_3PCNDmp$ (2NP)

To a solution of [P(μ-NTer)$_2$N] (ca. 30% purity, 70% Ter_2N_3H; 150 mg) in benzene (3 ml), a solution of CNDmp (28 mg, 0.213 mmol) in benzene (2 ml) was added. The initially yellow solution turned red. Various attempts of crystallization failed and the product could not be isolated.

^{31}P NMR (298 K, C_6D_6, 121.5 MHz): 167.3 (s). **UV/vis** (λ_{max}, nm): 490.

3. Crystallographic Data

Table S1. Crystallographic data of **2NAs**, **2PAs** and **3PAs**.

compound	**2NAs**	**2PAs**	**2'PAs**	**3PAs**
sum formula	$C_{72}H_{74}AsN_4$	$C_{65}H_{71}AsN_3P$	$C_{60}H_{62}AsN_3P$	$C_{65.25}H_{71.25}AsN_3P_2$
formular weight [g mol^{-1}]	1070.27	931.01	931.01	1034.36
colour	red	green	green	yellow
crystal system	triclinic	triclinic	triclinic	monoclinic
space group	*P*-1	*P*-1	*P*-1	$P2_1/c$
a [Å]	10.8398(18)	10.6316(9)	10.6707(5)	24.123(2)
b [Å]	16.340(3)	12.8458(10)	12.7933(6)	12.4043(9)
c [Å]	17.690(3)	20.4651(17)	20.5386(9)	39.140(3)
α [°]	85.439(6)	85.732(4)	85.861(3)	90
β [°]	75.150(5)	82.704(5)	82.563(3)	106.871(3)
γ [°]	82.111(6)	66.749(4)	67.099(2)	90
V [Å^3]	2996.9(9)	2546.3(4)	2560.3(2)	11207.6(15)
Z	2	2	2	8
$\rho_{calc.}$ [g cm^{-3}]	1.186	1.214	1.214	1.226
μ [mm^{-1}]	0.612	0.740	0.736	0.706
$\lambda_{MoK\alpha}$ [Å]	0.71073	0.71073	0.71073	0.71073
T [K]	173	123	123	173
measured reflexes	46291	20662	25244	72808
independent reflexes	10440	14001	14381	31138
reflexes $I > 2\sigma(I)$	5013	10531	8482	21139
$R_{int.}$	0.1198	0.0549	0.0667	0.0502
$2\Theta_{max.}$ [°]	50	60	60	60
F(000)	1134	982	982	4374
R_1 (R [$F^2 > 2\sigma(F^2)$])	0.0596	0.0576	0.0465	0.0511
wR_2 (all data)	0.1470	0.1486	0.1013	0.1204
GooF	0.965	1.008	0.877	1.005
parameter	685	613	625	1286
CCDC #	**1421413**	**1421414**	**1421415**	**1421416**

3.1. Numbering scheme of **2NAs**

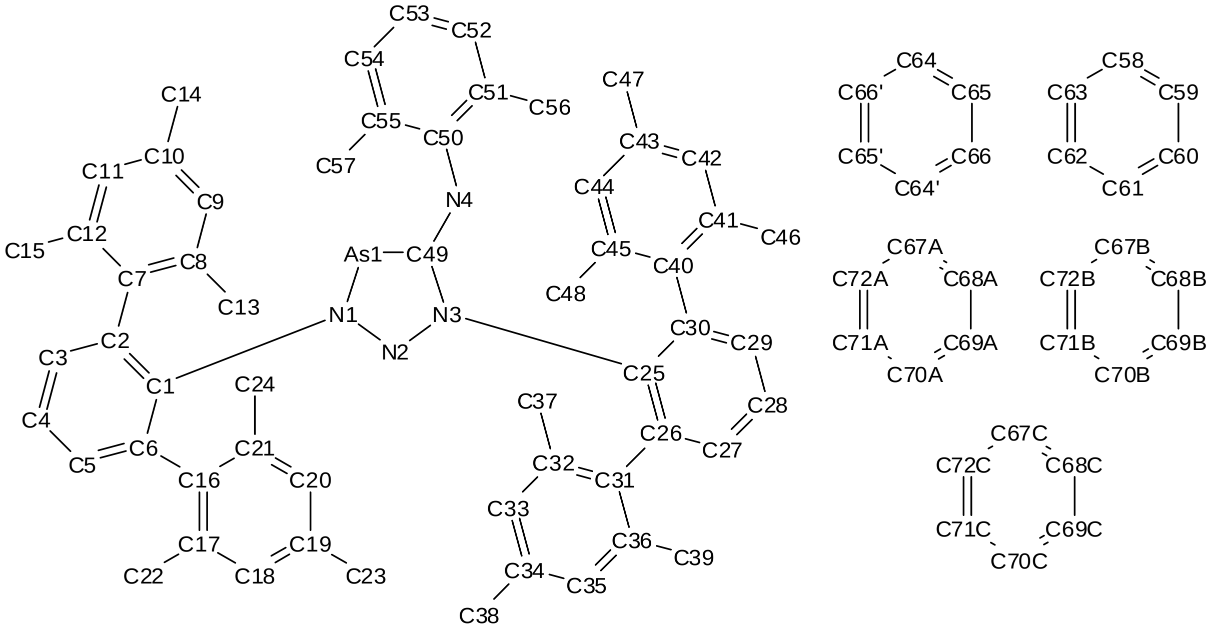

Selected bond lengths [Å] and angles [°] of **2**

**As1–N1**	1.875(3)	**N2–N1–As1**	119.4(2)
**As1–C49**	1.902(4)	**N1–N2–N3**	109.8(3)
**N1–N2**	1.316(4)	**N2–N3–C49**	119.4(3)
**N2–N3**	1.349(4)	**C49–N3–C25**	123.4(3)
**N3–C49**	1.428(5)	**C49–N4–C50**	116.3(4)
**N4–C49**	1.293(5)	**C49–As1–N1–N2**	1.8(3)
**N4–C50**	1.428(5)	**As1–N1–N2–N3**	–0.7(4)
**N1–As1–C49**	82.71(16)	**N1–N2–N3–C49**	–1.3(5)

3.2. Numbering scheme of **2PAs**

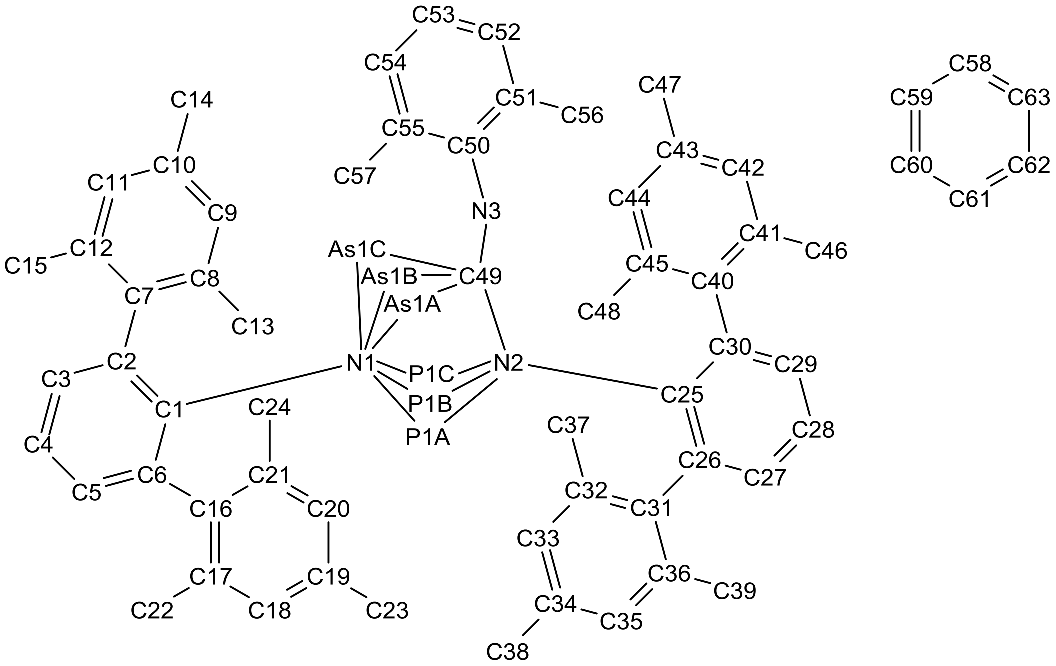

Selected bond lengths [Å] and angles [°] of **2PAs**

**As1B–N1**	1.8740(19)	**P1B–N1–As1B**	120.45(10)
**As1B–C49**	1.937(2)	**N1–As1B–C49**	88.51(8)
**P1B–N1**	1.6356(19)	**N1–P1B–N2**	97.69(9)
**P1B–N2**	1.6909(19)		

3.3. Numbering scheme of **2'PAs**

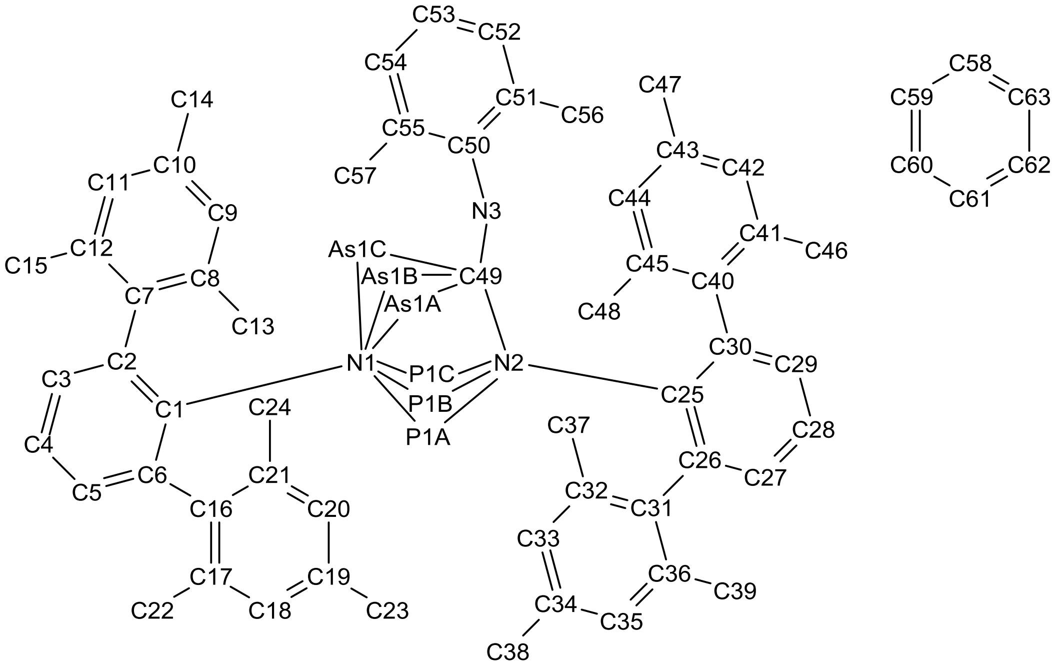

Selected bond lengths [Å] and angles [°] of **2'PAs**

**As1A–N1**	1.9696(14)	**N1–C1**	1.447(2)
**As1A–C49**	2.0105(17)	**N2–C49**	1.393(2)
**As1A–P1A**	2.2920(7)	**N2–C25**	1.441(2)
**P1A–N1**	1.6917(16)	**N3–C49**	1.252(2)
**P1A–N2**	1.8009(15)	**N3–C50**	1.422(2)
**N1–As1A–C49**	87.16(7)	**N2–P1A–As1A**	81.94(5)
**N1–P1A–N2**	95.12(7)	**P1A–N1–As1A**	77.10(6)
**N1–P1A–As1A**	56.89(5)	**N1–As1A–P1A**	46.01(5)

3.4. Numbering scheme of **3PAs**

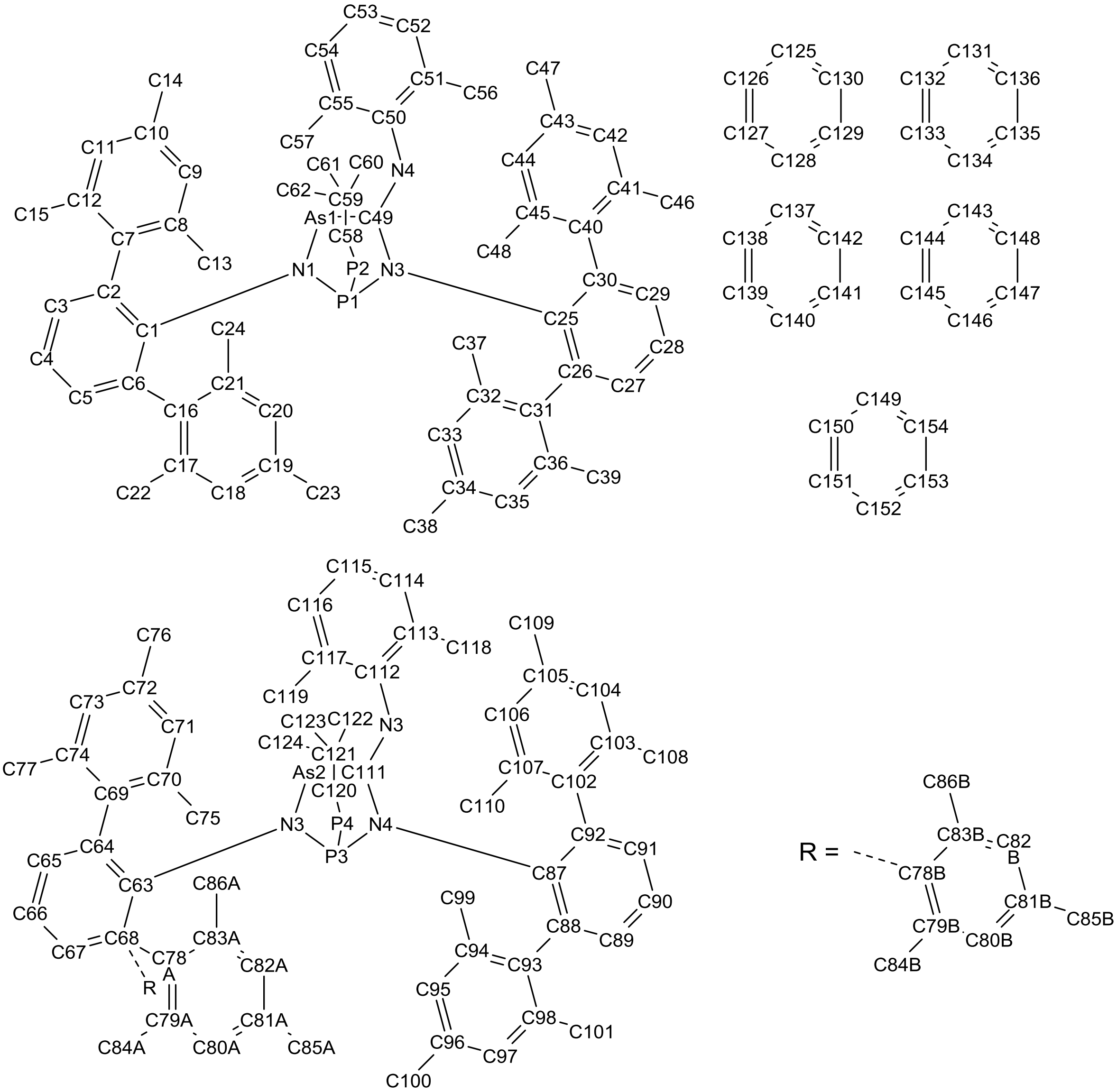

Selected bond lengths [Å] and angles [°] of **3**.

**As1–N1**	1.8950(18)	**As1–C58**	1.996(2)
**As1–C49**	2.007(2)	**N2–C25**	1.452(3)
**P1–N1**	1.7378(18)	**N3–C49**	1.270(3)
**P1–N2**	1.756(2)	**P1–P2**	2.2822(8)
**P2–C58**	1.673(2)	**N2–C49**	1.392(3)
**N3–C50**	1.415(3)	**N1–As1–C49**	88.96(8)
**C58–P2–P1**	96.42(8)	**N1–P1–N2**	93.94(9)
**C49–N2–P1**	117.97(14)	**P1–N1–As1**	106.81(9)

4. Computational Details

Utilizing the experimental structural data, all calculations were carried out with the Gaussian 09 package of molecular orbital programs.[10] The wave functions for the crystal structures were optimized with a 6-31G(d,p) basis set on the pbe1pbe level of density functional theory and the optimized structures were checked to be a minimum on the energy hypersurface. For Sb a relativistic pseudopotential was used, Sb: ECP46MDF 4 46.
ELF[9] and NBO/NRT[12–14] analyses were carried out to study the bonding, hybridization and polarization effects.

For **5** and **6** the ^{31}P NMR chemical shifts and coupling constants were calculated using the GIAO package implemented in Gaussian 09. The calculated absolute shifts (σ_{iso}) were referenced to the absolute chemical shift the standard (^{31}P: H_3PO_4, σ_{ref} =374.0604; ^{1}H, ^{13}C: $SiMe_4$, 31.665 and 196.4544 ppm, respecitvely), using the formula $\delta_{calc} = \sigma_{ref} - \sigma_{iso}$. Simulations of NMR spectra were performed with gNMR 5.06, which is obtainable free of charge from Peter H.M. Budzelaar via http://home.cc.umanitoba.ca/~budzelaa/gNMR/gNMR.html.

It should be emphasized that the computation was carried out for a single, isolated (gas-phase) molecule.

4.1. Optimized geometries

4.1.1. $Ter_2N_3PCNDmp$

P	0.30046800	-0.77023100	-1.68519700
N	0.57308300	0.75125700	-0.89061200
N	0.20281200	0.89971200	0.34856900
N	-0.33739900	-0.23952500	0.77558800
N	-0.73783700	-2.50243600	0.27678600
C	1.19267900	1.85654900	-1.58214300
C	2.45419100	1.64474900	-2.18242300
C	3.05655700	2.70349500	-2.86699800
H	4.03405000	2.53246700	-3.30828500
C	2.43968900	3.93946100	-2.97801500
H	2.92357800	4.75215600	-3.51151700
C	1.18540000	4.11645400	-2.41794000
H	0.66564700	5.06359800	-2.52892400
C	0.52795400	3.08879500	-1.72916100
C	3.22735500	0.36820400	-2.13503500
C	3.25618700	-0.48053800	-3.25755900
C	4.02705800	-1.63914000	-3.20030100
H	4.03591900	-2.30521700	-4.06064100
C	4.78229300	-1.96914400	-2.07440300
C	4.78063800	-1.08267900	-1.00026900
H	5.39061900	-1.30043000	-0.12582200
C	4.02336800	0.08897700	-1.01507100
C	2.47880700	-0.15908400	-4.50408600
H	1.41406500	-0.01831900	-4.28924700
H	2.56802400	-0.96644700	-5.23500600
H	2.83390100	0.76397100	-4.97511300
C	5.56722200	-3.24954700	-2.02392500
H	6.00814200	-3.48804700	-2.99654200
H	4.92268500	-4.09169300	-1.74550400
H	6.37430300	-3.19597900	-1.28799100
C	4.08772000	1.05092000	0.13448900
H	4.27331700	2.07228600	-0.21427300
H	4.88592600	0.77378900	0.82410900
H	3.15498900	1.07058500	0.70793200
C	-0.86356800	3.43152200	-1.30451000
C	-1.94875300	3.05766600	-2.12040100
C	-3.20570900	3.60198700	-1.85904600
H	-4.03872900	3.32155400	-2.50077100
C	-3.42003100	4.50635700	-0.81933100
C	-2.33327400	4.85440300	-0.02068300
H	-2.47573700	5.55770600	0.79756900
C	-1.05607200	4.34103100	-0.25043600
C	-1.76501500	2.13464700	-3.29248400
H	-0.97752600	2.49462700	-3.96351800
H	-2.68967800	2.04997000	-3.86863300
H	-1.47744400	1.12705200	-2.97161400
C	-4.79062900	5.06032500	-0.54912400
H	-4.73888700	6.02919500	-0.04472900
H	-5.36856400	4.38756600	0.09696500
H	-5.36000600	5.18771900	-1.47446500
C	0.09661500	4.80954900	0.59460700
H	0.65681100	5.60965100	0.09592100
H	0.80406800	4.00196600	0.79710000
H	-0.25878500	5.20461700	1.55009700
C	-0.78136800	-0.33740600	2.14206700
C	0.12069300	-0.11108000	3.19849700
C	-0.33888400	-0.25723000	4.51376000
H	0.36707100	-0.08000500	5.32006300
C	-1.64214800	-0.63032300	4.79356300
H	-1.97502900	-0.74279700	5.82114100
C	-2.51727800	-0.84845400	3.74175200
H	-3.55162900	-1.11524300	3.93596100
C	-2.11819800	-0.70590900	2.41002800
C	1.56457300	0.26697100	3.10735700
C	1.93683200	1.60001400	3.35966800

C	3.28430500	1.90255300	3.55417200
H	3.56683800	2.93423100	3.75548800
C	4.27024100	0.91673200	3.52817700
C	3.87927000	-0.39473800	3.26518400
H	4.63211500	-1.18052100	3.23939400
C	2.54351900	-0.73918800	3.05259100
C	0.89650100	2.67731100	3.48595100
H	0.35332900	2.59373800	4.43454400
H	0.15370000	2.60806800	2.68670200
H	1.35469600	3.66927800	3.45084200
C	5.71592900	1.26686600	3.74429500
H	5.82580600	2.08232000	4.46531100
H	6.18890900	1.59621100	2.81062600
H	6.28441000	0.40834600	4.11277800
C	2.15753500	-2.17502500	2.84417700
H	3.03276700	-2.78471900	2.60483000
H	1.42596500	-2.29053200	2.04037500
H	1.69595600	-2.59192100	3.74763500
C	-3.19820100	-0.87980900	1.39616100
C	-3.73805500	0.26023800	0.77734100
C	-4.83058900	0.11447100	-0.07951400
H	-5.24049700	1.00190400	-0.55821900
C	-5.42151800	-1.12504300	-0.31211600
C	-4.90151500	-2.23363800	0.35498800
H	-5.35989400	-3.20905000	0.20359800
C	-3.80503700	-2.13797400	1.21106000
C	-3.20998200	1.63111000	1.08586900
H	-3.91930900	2.39557700	0.76676500
H	-2.26305900	1.83949400	0.57737700
H	-3.03463300	1.75458900	2.15915900
C	-6.57599500	-1.26927000	-1.26338000
H	-7.12660100	-0.33031900	-1.37120200
H	-7.27760400	-2.03896400	-0.92810300
H	-6.22800200	-1.56119800	-2.26177200
C	-3.32472000	-3.36627500	1.93339100
H	-3.76337900	-3.43610800	2.93629700
H	-2.23802200	-3.35525700	2.03429000
H	-3.61065200	-4.26842000	1.38622800
C	-0.35909000	-1.33709500	-0.12209000
C	-0.84024700	-3.55479400	-0.63867700
C	-1.64671600	-3.50517700	-1.80240400
C	-1.70862800	-4.63240200	-2.62958900
H	-2.33119600	-4.58365200	-3.52069500
C	-1.02433300	-5.79887900	-2.33035600
H	-1.09308100	-6.66212800	-2.98605600
C	-0.26886600	-5.85701200	-1.16268900
H	0.25931400	-6.77132700	-0.90164400
C	-0.17357700	-4.76248200	-0.31132900
C	-2.49048000	-2.31484200	-2.16585800
H	-2.70878600	-1.68647000	-1.30144900
H	-3.44386300	-2.64842800	-2.58782400
H	-2.00486100	-1.68213600	-2.92099600
C	0.65200300	-4.85306900	0.93766700
H	1.57947700	-4.27707900	0.84512000
H	0.92263900	-5.89134300	1.15009600
H	0.11481300	-4.44474800	1.79910400

4.1.2. $Ter_2N_3AsCNDmp$

0 1

As	-1.06624800	1.24586400	-0.95845900
N	-0.84880100	-0.59435500	-0.65316900
N	0.01969700	-0.97924400	0.23096400
N	0.62170600	0.06748800	0.78267100
N	0.79404800	2.40138700	0.92316000
C	-1.54216000	-1.59832700	-1.40129600
C	-2.88600000	-1.35413300	-1.77063800

C	-3.60951900	-2.37140000	-2.39467500
H	-4.64623200	-2.17379100	-2.65096700
C	-3.02286700	-3.58772200	-2.70929100
H	-3.59635700	-4.36834100	-3.19985600
C	-1.67456000	-3.76423400	-2.44966500
H	-1.17227600	-4.66770300	-2.78152100
C	-0.90035800	-2.78375600	-1.81501800
C	-3.58921300	-0.04400900	-1.62564900
C	-3.62825700	0.83528100	-2.72472200
C	-4.30325600	2.04748500	-2.58770000
H	-4.31099800	2.74025000	-3.42654900
C	-4.94919300	2.40352200	-1.40551600
C	-4.92885300	1.49747500	-0.34502900
H	-5.43804400	1.75033100	0.58271600
C	-4.26612100	0.27748600	-0.43762700
C	-2.86297700	0.55239300	-3.98686500
H	-1.79033200	0.69558800	-3.80172700
H	-3.15805200	1.23814800	-4.78488000
H	-3.00196900	-0.47252000	-4.34135300
C	-5.67991000	3.71066700	-1.28618200
H	-6.75303200	3.58005700	-1.47045800
H	-5.30863200	4.44278200	-2.00803400
H	-5.57121300	4.13703600	-0.28476800
C	-4.25661700	-0.67824900	0.71399200
H	-4.65973500	-1.65650800	0.43027500
H	-4.84505800	-0.29441100	1.55046100
H	-3.23655600	-0.84722600	1.07094000
C	0.56632500	-3.06583200	-1.82271800
C	1.40182400	-2.33778100	-2.69419800
C	2.68988300	-2.81053400	-2.94189000
H	3.32110800	-2.25978000	-3.63665800
C	3.18652700	-3.96465400	-2.33744700
C	2.36292400	-4.63430500	-1.43572700
H	2.73705600	-5.52259800	-0.93000300
C	1.05908200	-4.21147800	-1.17258500
C	0.92165200	-1.09424900	-3.38921200
H	0.06495800	-1.30568400	-4.03986900
H	1.71614300	-0.66462500	-4.00398200
H	0.59907400	-0.32656700	-2.67638500
C	4.58602200	-4.43932200	-2.61058200
H	4.67558800	-5.52091100	-2.47541300
H	5.30561400	-3.96673400	-1.92999600
H	4.89809200	-4.19667600	-3.63056600
C	0.20498100	-5.00243800	-0.21775900
H	-0.44295700	-5.71528600	-0.74069000
H	-0.44376000	-4.35184500	0.37486300
H	0.83331300	-5.57840100	0.46689700
C	1.41890300	-0.12969200	1.96446500
C	0.84476200	-0.73652500	3.09543100
C	1.58460900	-0.75991600	4.28436900
H	1.12950000	-1.21765100	5.15803800
C	2.85301000	-0.20866600	4.36247800
H	3.40221700	-0.22507800	5.29926300
C	3.42405000	0.33661000	3.22206300
H	4.43802600	0.72386800	3.24720300
C	2.73424400	0.38024500	2.00861100
C	-0.48491000	-1.41535500	3.16288300
C	-0.50427600	-2.82270500	3.22158700
C	-1.71013100	-3.47610300	3.47212300
H	-1.71930000	-4.56370100	3.51254400
C	-2.89289800	-2.77414500	3.69961000
C	-2.84723100	-1.38341000	3.64102500
H	-3.75732400	-0.81464000	3.82046600
C	-1.67117200	-0.68875100	3.35190800
C	0.75493800	-3.62588000	3.04113600
H	1.44934100	-3.48124400	3.87554900
H	1.28829500	-3.33239600	2.13131800
H	0.52654500	-4.69276200	2.97698500
C	-4.18340600	-3.49586000	3.96942700

H	-4.00983000	-4.45642500	4.46292400
H	-4.72239100	-3.70135800	3.03657100
H	-4.84797700	-2.90278300	4.60439400
C	-1.69627000	0.80771500	3.22445800
H	-2.60934700	1.22518400	3.65739600
H	-1.66134700	1.10181100	2.16810700
H	-0.83505500	1.27361100	3.71060600
C	3.49865200	0.84022900	0.81520200
C	3.90165800	-0.13059100	-0.11990200
C	4.67127300	0.25988000	-1.21583600
H	4.97788800	-0.49621000	-1.93626000
C	5.08061400	1.58037000	-1.39175200
C	4.73229500	2.50855300	-0.41239700
H	5.07147700	3.53808700	-0.51083500
C	3.95967700	2.16474600	0.69804500
C	3.59431600	-1.58515800	0.09820700
H	4.21269400	-2.20654800	-0.55060800
H	2.54987300	-1.83318900	-0.11746000
H	3.79064700	-1.87436800	1.13563800
C	5.87341300	1.99362400	-2.59988800
H	6.44128000	1.15490300	-3.01305400
H	6.57713000	2.79665200	-2.36143400
H	5.21458100	2.36546400	-3.39404000
C	3.68899500	3.20450900	1.74970400
H	4.49183300	3.22109100	2.49749900
H	2.74378800	3.01167600	2.25731700
H	3.63899500	4.20079300	1.30206900
C	0.29455400	1.37054700	0.33928900
C	0.47718500	3.66833900	0.42584400
C	0.76094400	4.06952700	-0.90343100
C	0.44216900	5.37470600	-1.29325500
H	0.66499500	5.67854000	-2.31398700
C	-0.11960700	6.28468600	-0.41150400
H	-0.35206000	7.29455700	-0.73717000
C	-0.35842000	5.89541000	0.90334700
H	-0.78139000	6.60407700	1.61192700
C	-0.06120800	4.60834400	1.33793100
C	1.44521200	3.16894900	-1.89416400
H	2.08958400	2.43935100	-1.39912200
H	2.06111700	3.76200500	-2.57730200
H	0.72420300	2.60836400	-2.50593500
C	-0.33018300	4.20701300	2.75853800
H	-1.22397300	3.57741900	2.83195400
H	-0.48899800	5.08666100	3.38890000
H	0.49814400	3.62041700	3.16651200

4.1.3. $Ter_2N_3SbCNDmp$

Sb	-0.83355000	1.17602000	-1.49681100
N	-0.82682700	-0.74018000	-0.59494400
N	-0.10742300	-0.93272300	0.46382500
N	0.55576900	0.14954900	0.86266700
N	1.13226900	2.39647500	0.54066400
C	-1.54961200	-1.87728700	-1.09362600
C	-2.89097800	-1.67912100	-1.50367600
C	-3.66533800	-2.77971700	-1.87623300
H	-4.69895500	-2.60131800	-2.15887100
C	-3.13631100	-4.05996700	-1.90274400
H	-3.74886400	-4.90922600	-2.19020300
C	-1.79160600	-4.22458200	-1.62006900
H	-1.33200000	-5.20205300	-1.73068500
C	-0.96431800	-3.15820100	-1.23778500
C	-3.55840100	-0.35131500	-1.65776200
C	-3.67655000	0.19715000	-2.95128600
C	-4.35006800	1.40885300	-3.10815500
H	-4.42347200	1.84262300	-4.10333100
C	-4.93111400	2.07424600	-2.02980300
C	-4.85231500	1.47976400	-0.76996100
H	-5.32559000	1.96718500	0.08005100

C	-4.18184800	0.27690300	-0.56536300
C	-3.09219800	-0.48928600	-4.15715900
H	-2.04780200	-0.77565900	-3.99729500
H	-3.13083700	0.16689400	-5.03022500
H	-3.63831700	-1.40735000	-4.40092100
C	-5.61954000	3.39695200	-2.21598000
H	-6.47987100	3.49730300	-1.54754200
H	-5.96894100	3.52578800	-3.24418700
H	-4.93710200	4.22590300	-1.99499000
C	-4.12641600	-0.34338300	0.79624800
H	-4.50500300	-1.37116300	0.78189400
H	-4.71661900	0.23185700	1.51186600
H	-3.10114800	-0.38842600	1.17775100
C	0.48628900	-3.52551000	-1.20386500
C	1.32830500	-3.09395200	-2.25295700
C	2.59231200	-3.66576500	-2.38870000
H	3.22471700	-3.33843300	-3.21167700
C	3.05575900	-4.66002200	-1.52760200
C	2.21419400	-5.06871300	-0.49799800
H	2.54885900	-5.84463400	0.18847200
C	0.93666800	-4.53060000	-0.32720700
C	0.86995300	-2.09392300	-3.27598800
H	-0.08771400	-2.38990100	-3.71856700
H	1.60155300	-2.00460800	-4.08302200
H	0.73076900	-1.09606100	-2.84433200
C	4.43043000	-5.24523900	-1.68945700
H	4.47954900	-6.26639100	-1.30080300
H	5.17841500	-4.65436500	-1.14567900
H	4.73497100	-5.26523200	-2.73999200
C	0.06763400	-5.09880000	0.76316600
H	-0.34527200	-6.07204900	0.47049100
H	-0.77195500	-4.44693800	1.00468700
H	0.65134600	-5.26060500	1.67464700
C	1.23010900	0.06642400	2.14041400
C	0.47460500	-0.18243200	3.30332600
C	1.13440000	-0.23540700	4.53773400
H	0.53860900	-0.43173700	5.42473800
C	2.49833800	-0.02190100	4.64269500
H	2.99070700	-0.06114500	5.61002100
C	3.22442600	0.24408800	3.49282500
H	4.29737300	0.40290800	3.54734300
C	2.62131500	0.29197700	2.23178600
C	-1.00705800	-0.36266500	3.40322000
C	-1.54017700	-1.65540300	3.54878500
C	-2.88525600	-1.80188100	3.88827200
H	-3.29180200	-2.80538500	4.00054900
C	-3.71023900	-0.70082900	4.11628200
C	-3.15830700	0.57152900	3.97685700
H	-3.78048100	1.44580200	4.16007500
C	-1.82112600	0.76177200	3.62515500
C	-0.66024400	-2.86338300	3.40833400
H	0.01955300	-2.95796700	4.26348200
H	-0.03916400	-2.79426000	2.51158900
H	-1.25600700	-3.77841500	3.35431700
C	-5.15829300	-0.88279000	4.47584900
H	-5.31074300	-1.78280900	5.07898900
H	-5.77845000	-0.98659000	3.57684600
H	-5.54109100	-0.02708600	5.03947100
C	-1.24752000	2.14781900	3.56293800
H	-2.04086800	2.89990200	3.54530500
H	-0.61551600	2.28858000	2.68269000
H	-0.61577300	2.34874400	4.43697200
C	3.55982500	0.50211600	1.09120900
C	3.92759700	-0.60571900	0.30928400
C	4.89054900	-0.44514800	-0.68864100
H	5.16593100	-1.30752200	-1.29318700
C	5.52548200	0.77605000	-0.90532900
C	5.19085900	1.84405900	-0.07372300
H	5.69469000	2.79969400	-0.20660000

C	4.22707900	1.73200100	0.92899800
C	3.36754000	-1.96676800	0.60123100
H	3.90929400	-2.73284400	0.04651800
H	2.31073700	-2.05572300	0.33051800
H	3.44586400	-2.19870800	1.66877500
C	6.53477400	0.94126600	-2.00690100
H	7.03335000	-0.00445500	-2.23918200
H	7.30195700	1.67443600	-1.74064600
H	6.05625100	1.29251100	-2.92946400
C	3.96343400	2.90640800	1.83020700
H	4.55209800	2.83727600	2.75323800
H	2.90718700	2.95863400	2.09970600
H	4.24014200	3.84056400	1.33389700
C	0.50872600	1.35273100	0.11778900
C	1.15964100	3.50998900	-0.30776300
C	1.75025400	3.46976900	-1.59706600
C	1.75408400	4.63614100	-2.37180600
H	2.21374800	4.59853400	-3.35741900
C	1.21754200	5.82597800	-1.90662700
H	1.23796600	6.71858800	-2.52540100
C	0.67578300	5.86856900	-0.62484000
H	0.26802200	6.79950800	-0.23732600
C	0.64463700	4.73619200	0.18209000
C	2.43932100	2.25396100	-2.15697600
H	2.65199100	1.50707900	-1.39177600
H	3.39068300	2.54543600	-2.61419600
H	1.83595400	1.77004400	-2.93581700
C	0.05180600	4.80939300	1.55779300
H	-0.90929100	4.28658900	1.60359000
H	-0.11689800	5.84877300	1.85339400
H	0.70476900	4.33470200	2.29668200

4.1.4. $Ter_2N_2PAsCNDmp$

0 1

As	-0.71111400	0.90506400	-1.40253100
N	-0.90804400	-0.85617100	-0.77096900
P	-0.18120200	-1.29626200	0.65168900
N	0.55985400	0.19196200	0.95450200
N	1.05129900	2.38511300	0.29480400
C	-1.60394400	-1.82772700	-1.55969500
C	-2.87103900	-1.50550600	-2.09600000
C	-3.55383100	-2.45960400	-2.85139100
H	-4.53312500	-2.19739500	-3.24173300
C	-3.00757900	-3.71098800	-3.09893900
H	-3.55008800	-4.44319400	-3.68934300
C	-1.74881300	-4.00518200	-2.59989400
H	-1.28495000	-4.96313200	-2.81685000
C	-1.02154100	-3.08272100	-1.83779600
C	-3.52063900	-0.18178700	-1.88526400
C	-3.51205100	0.78055100	-2.91163600
C	-4.07995400	2.02828200	-2.66199200
H	-4.04485500	2.78567200	-3.44223400
C	-4.67063400	2.33877400	-1.43780600
C	-4.71259300	1.34904000	-0.45702700
H	-5.19509000	1.56135800	0.49513500
C	-4.14789400	0.09158100	-0.65974100
C	-2.82079000	0.52167900	-4.22186500
H	-1.73768200	0.44046000	-4.06462600
H	-2.99649700	1.34142400	-4.92273200
H	-3.15177000	-0.40920500	-4.69091600
C	-5.28758600	3.68832700	-1.20105500
H	-6.33391800	3.70773900	-1.52918400
H	-4.75795400	4.47001900	-1.75276200
H	-5.27307900	3.95327700	-0.14011900
C	-4.25108600	-0.96634900	0.40184900
H	-4.60050400	-1.91457200	-0.01934600
H	-4.94707200	-0.66099900	1.18581000

H	-3.28687100	-1.16027700	0.88357400
C	0.36146000	-3.51046400	-1.46223000
C	1.46019100	-3.01818500	-2.19546900
C	2.71633000	-3.58437100	-1.98908800
H	3.55930100	-3.20536000	-2.56334200
C	2.91977500	-4.61982000	-1.07586900
C	1.82682300	-5.06538600	-0.33618700
H	1.96832400	-5.85447400	0.39954800
C	0.54802800	-4.53556500	-0.51681700
C	1.28299900	-1.91433500	-3.19940900
H	0.63502500	-2.22999600	-4.02552100
H	2.24482600	-1.60867500	-3.61807700
H	0.81554300	-1.03427200	-2.74336000
C	4.27843900	-5.23932500	-0.90532300
H	4.49798100	-5.93667000	-1.72247000
H	4.34960200	-5.79831600	0.03159600
H	5.06881600	-4.48188600	-0.90905700
C	-0.59972600	-5.06178800	0.30126600
H	-1.32636900	-5.60335500	-0.31338700
H	-1.14709900	-4.24910300	0.79146300
H	-0.24197500	-5.74389100	1.07670600
C	1.18621800	0.38618200	2.23741500
C	0.39666700	0.28238800	3.39997800
C	0.98945100	0.55239500	4.63962400
H	0.36903100	0.48968600	5.52899100
C	2.32611600	0.90086500	4.74082800
H	2.76609400	1.11481500	5.71041800
C	3.10204600	0.95025900	3.59149700
H	4.16187100	1.17732000	3.65690900
C	2.56198400	0.68958600	2.32983100
C	-1.04275200	-0.12804600	3.45223400
C	-1.35822100	-1.38879200	4.00406000
C	-2.69159100	-1.70829500	4.25402300
H	-2.92628400	-2.68202600	4.67974000
C	-3.72521000	-0.80841300	3.99695100
C	-3.39350700	0.42145800	3.43448200
H	-4.18370400	1.13814900	3.21832300
C	-2.07627400	0.77288100	3.13621800
C	-0.28716400	-2.39542400	4.32787400
H	0.33582300	-2.07696600	5.17006000
H	0.38851900	-2.54884900	3.47939900
H	-0.73165100	-3.36030600	4.58536300
C	-5.15444200	-1.17204800	4.28500300
H	-5.23824600	-1.77981500	5.19083400
H	-5.58693700	-1.75565600	3.46297800
H	-5.77506400	-0.28111500	4.41553500
C	-1.79477900	2.08878200	2.47291500
H	-2.59848300	2.80515700	2.66475100
H	-1.71647500	1.95753500	1.38687500
H	-0.85033600	2.52147700	2.80949600
C	3.51639500	0.67147700	1.18337700
C	3.88357800	-0.55863000	0.60998100
C	4.83894300	-0.57583600	-0.40956700
H	5.11743600	-1.53284100	-0.84722500
C	5.45802400	0.58612900	-0.85878700
C	5.11928500	1.78783900	-0.23671200
H	5.60675600	2.70736600	-0.55480200
C	4.17052200	1.85515700	0.78163100
C	3.32002500	-1.86205600	1.10093800
H	4.01955600	-2.67969600	0.91031300
H	2.38733000	-2.12476400	0.58696600
H	3.11182900	-1.83524800	2.17403400
C	6.45406500	0.55434000	-1.98365600
H	6.86580800	-0.44878000	-2.12667600
H	7.28683800	1.24062200	-1.80158800
H	5.98794700	0.85661100	-2.92926400
C	3.89152700	3.17924500	1.43691900
H	4.47329400	3.29850900	2.35903100
H	2.83240000	3.26986500	1.68326500

H	4.16192300	4.00147000	0.76927000
C	0.47777900	1.26411700	0.03063700
C	1.03289500	3.40699800	-0.66130200
C	1.60677700	3.26298500	-1.94952100
C	1.59956300	4.36008000	-2.81658000
H	2.04398300	4.24164600	-3.80269100
C	1.06536200	5.58390500	-2.44458200
H	1.07689000	6.42269200	-3.13470500
C	0.53678000	5.72923400	-1.16593900
H	0.13224100	6.68850400	-0.85062600
C	0.52025700	4.66721900	-0.26770500
C	2.25774100	1.99064200	-2.41331100
H	2.61703700	1.38724600	-1.57760000
H	3.11003000	2.21799300	-3.06168100
H	1.55605100	1.37290700	-2.98984400
C	-0.04921500	4.85511800	1.10731100
H	-1.03893100	4.39418800	1.19545200
H	-0.15402400	5.91802100	1.34314000
H	0.58480700	4.38435400	1.86450600

4.1.5. $Ter_2N_3PSbCNDmp$

0 1

Sb	0.86514700	-1.32233600	-1.25685900
N	1.07247500	0.70866000	-0.66807700
P	0.14209400	1.30391200	0.55958600
N	-0.77988200	-0.06647000	0.93820900
N	-1.39672400	-2.29259200	0.55901600
C	1.95488000	1.62960100	-1.31895000
C	3.28383900	1.24137700	-1.61760600
C	4.16179400	2.16983100	-2.18214300
H	5.18032700	1.84834500	-2.38135600
C	3.76080800	3.46142100	-2.48656400
H	4.45723100	4.17046700	-2.92392300
C	2.44232600	3.81713300	-2.25536500
H	2.08520800	4.80293700	-2.53995300
C	1.52127200	2.92699800	-1.68909000
C	3.83319500	-0.13064300	-1.41198500
C	4.01507900	-0.96766700	-2.53082000
C	4.57963900	-2.22911700	-2.34273700
H	4.69979200	-2.88365600	-3.20367700
C	4.99043100	-2.67332400	-1.08686500
C	4.84619100	-1.80706200	-0.00360700
H	5.18582400	-2.12372900	0.98065800
C	4.27556100	-0.54406700	-0.14323300
C	3.60375000	-0.53739700	-3.91395200
H	2.59265200	-0.11782600	-3.92499700
H	3.62387200	-1.38598900	-4.60241300
H	4.27177700	0.23436000	-4.31245800
C	5.55839900	-4.05216700	-0.90178900
H	6.29865700	-4.07566000	-0.09657800
H	6.03636700	-4.41538300	-1.81605200
H	4.76789200	-4.76525000	-0.64001500
C	4.14426900	0.36317400	1.04401900
H	4.55286000	1.35754500	0.83481700
H	4.66580700	-0.04997400	1.91048000
H	3.09565700	0.49722500	1.32865100
C	0.11712600	3.44625600	-1.64181900
C	-0.79429900	3.05132300	-2.64218400
C	-2.01413700	3.71959300	-2.74811300
H	-2.70607200	3.42071100	-3.53319900
C	-2.36051200	4.76824500	-1.89708300
C	-1.45190600	5.13213600	-0.90422600
H	-1.70172100	5.94578300	-0.22577200
C	-0.21646900	4.49876200	-0.76641700
C	-0.45264800	1.96046700	-3.61756200
H	0.49230400	2.16986000	-4.13139200
H	-1.23532700	1.85379000	-4.37290800
H	-0.33675100	0.99280600	-3.11548900

C	-3.68264700	5.47035700	-2.03085700
H	-3.60311200	6.52742500	-1.76051700
H	-4.43890400	5.02489400	-1.37250500
H	-4.06506500	5.40851000	-3.05364100
C	0.74160200	4.96707200	0.29492800
H	1.59165500	5.50804800	-0.13522100
H	1.15764200	4.12813200	0.86259200
H	0.24096800	5.63828700	0.99782000
C	-1.63058700	0.04245100	2.10474100
C	-1.04433100	0.34444200	3.35384700
C	-1.85815700	0.40823500	4.49100600
H	-1.38825800	0.62972800	5.44519300
C	-3.22367800	0.18969500	4.41462100
H	-3.84118200	0.23859200	5.30675800
C	-3.79373400	-0.07033000	3.17771700
H	-4.86804700	-0.20269300	3.08973700
C	-3.02862200	-0.14029600	2.00907300
C	0.40619700	0.62239200	3.59320000
C	0.82841800	1.94789800	3.82714500
C	2.15068200	2.18035100	4.20555500
H	2.47363600	3.20517900	4.37870100
C	3.05927400	1.13883600	4.39078200
C	2.61260900	-0.16502000	4.18118200
H	3.29841000	-0.99544700	4.33819700
C	1.30391000	-0.44324800	3.78633700
C	-0.12490600	3.10637600	3.71676000
H	-0.87917100	3.07927600	4.51072900
H	-0.66936600	3.09956500	2.76680200
H	0.41019500	4.05652300	3.79454100
C	4.48194600	1.41679200	4.78730200
H	4.55540300	2.31016300	5.41438800
H	5.11093400	1.58656600	3.90482100
H	4.91449200	0.57729300	5.33924500
C	0.84520100	-1.86389700	3.63576300
H	1.69104300	-2.55524300	3.67251400
H	0.31044100	-2.02238600	2.69640200
H	0.15132200	-2.13676200	4.44010200
C	-3.80354500	-0.31511100	0.74595000
C	-3.97684400	0.78305300	-0.11486500
C	-4.80132900	0.64777000	-1.23487500
H	-4.92696700	1.50322300	-1.89627800
C	-5.48469700	-0.53378600	-1.50722200
C	-5.33843000	-1.59321800	-0.61174400
H	-5.87766700	-2.52118200	-0.79334500
C	-4.51735200	-1.50870500	0.51161900
C	-3.35264800	2.11735800	0.17656200
H	-3.89382400	2.91461100	-0.33712000
H	-2.31266500	2.17407600	-0.16711500
H	-3.35538800	2.33818000	1.24791600
C	-6.34245600	-0.67228400	-2.73354300
H	-6.67948300	0.30187800	-3.09952300
H	-7.22651100	-1.28660400	-2.53708600
H	-5.78790400	-1.15405300	-3.54820800
C	-4.43277100	-2.68033300	1.45050300
H	-5.12185900	-2.56780000	2.29621500
H	-3.41972600	-2.78338300	1.84391200
H	-4.69829000	-3.60565300	0.93210900
C	-0.67636000	-1.28369000	0.21598800
C	-1.31515700	-3.44282000	-0.23827500
C	-1.75688900	-3.46562200	-1.58513000
C	-1.66470800	-4.66428400	-2.30265600
H	-2.00857300	-4.67467800	-3.33499500
C	-1.17515900	-5.82589000	-1.72689700
H	-1.11929900	-6.74491600	-2.30343000
C	-0.78060400	-5.80617100	-0.39201000
H	-0.41383800	-6.71462200	0.08055300
C	-0.84731900	-4.63873100	0.36120300
C	-2.38098300	-2.28128900	-2.27348700
H	-2.59806900	-1.46624600	-1.58357700

```
 H                 -3.32405100   -2.58012800   -2.74403700
 H                 -1.72710000   -1.89092200   -3.06242600
 C                 -0.41496200   -4.64448100    1.79767800
 H                  0.56246700   -4.16496600    1.91873700
 H                 -0.33331200   -5.66785600    2.17502400
 H                 -1.11961300   -4.09019200    2.42474300
```

4.1.6. $Ter_2N_2As_2CNDmp$

```
0 1
 As                -0.03792700    1.70783800   -0.69898600
 N                 -1.22941800    0.26868400   -0.78270100
 As                -0.88809700   -1.22560100    0.14704000
 N                  0.70159300   -0.56161000    0.78080500
 N                  2.25214800    1.20554100    0.84310600
 C                 -2.38357800    0.32606900   -1.61431900
 C                 -3.12521200    1.52411200   -1.73122300
 C                 -4.26100300    1.54683600   -2.54279900
 H                 -4.82427000    2.47378000   -2.60465200
 C                 -4.66980700    0.42666100   -3.25217800
 H                 -5.55337500    0.46418600   -3.88208700
 C                 -3.92363900   -0.73866900   -3.15514600
 H                 -4.20464600   -1.62050500   -3.72401800
 C                 -2.78576500   -0.81187500   -2.34716400
 C                 -2.74453800    2.77047000   -1.01168000
 C                 -2.12312300    3.82616700   -1.70429400
 C                 -1.69998100    4.93989800   -0.98356400
 H                 -1.18212900    5.73915000   -1.50959400
 C                 -1.87543500    5.03526800    0.39701000
 C                 -2.54062600    4.00006500    1.05133800
 H                 -2.70780600    4.06784100    2.12448600
 C                 -2.97583600    2.86561500    0.36988300
 C                 -1.76014100    3.69954100   -3.15702800
 H                 -0.93853200    2.97659300   -3.25288700
 H                 -1.41488700    4.65501000   -3.55943500
 H                 -2.58764600    3.34055100   -3.77409500
 C                 -1.32920600    6.21174700    1.15449300
 H                 -1.54965400    7.15542200    0.64557300
 H                 -0.23887700    6.13497600    1.24021300
 H                 -1.74081200    6.26554100    2.16595000
 C                 -3.66437900    1.75000700    1.10012700
 H                 -4.63930000    1.52113800    0.65618600
 H                 -3.81793400    2.00569500    2.15062700
 H                 -3.06945200    0.83099100    1.06089800
 C                 -2.02413600   -2.09531000   -2.37280700
 C                 -0.81940400   -2.16427800   -3.10243100
 C                 -0.19846500   -3.40107400   -3.25548000
 H                  0.71749500   -3.45764500   -3.84017000
 C                 -0.70846300   -4.56173900   -2.67161600
 C                 -1.87288400   -4.45951300   -1.91251100
 H                 -2.27436500   -5.34898200   -1.43119400
 C                 -2.54310700   -3.24718900   -1.75240100
 C                 -0.21205500   -0.92800800   -3.70045300
 H                 -0.89267000   -0.44743100   -4.41184200
 H                  0.72147100   -1.16044500   -4.21817500
 H                  0.00508400   -0.19139400   -2.91755200
 C                  0.00045600   -5.87719000   -2.82856600
 H                 -0.67841000   -6.71955500   -2.67045100
 H                  0.81699400   -5.97325400   -2.10277400
 H                  0.44012100   -5.97689600   -3.82540500
 C                 -3.74993100   -3.16782200   -0.85802100
 H                 -4.60493400   -2.69263400   -1.34605700
 H                 -3.51387700   -2.56858700    0.03234800
 H                 -4.05348000   -4.16194700   -0.52039700
 C                  1.37192500   -1.32529500    1.78757700
 C                  0.62212800   -1.75393100    2.90481500
 C                  1.26561000   -2.42821100    3.94697200
 H                  0.67459300   -2.73156600    4.80649100
 C                  2.62236100   -2.70445200    3.89195000
```

```
 H                 3.11380200   -3.22168500    4.71059100
 C                 3.33571500   -2.34286500    2.75906300
 H                 4.38480700   -2.60911700    2.67053300
 C                 2.73927800   -1.66939300    1.68798200
 C                -0.85626600   -1.58121000    3.05473900
 C                -1.69002200   -2.71321600    2.91679400
 C                -3.04766400   -2.59724100    3.20765100
 H                -3.68665400   -3.46997600    3.08894500
 C                -3.60599200   -1.39642800    3.64495400
 C                -2.77056500   -0.28567700    3.74596400
 H                -3.18872600    0.66433300    4.07102000
 C                -1.41406300   -0.34479500    3.42935800
 C                -1.15507500   -4.00480400    2.36211000
 H                -0.28064100   -4.36906000    2.90718600
 H                -0.84411300   -3.85206000    1.31923800
 H                -1.92153200   -4.78372600    2.37296200
 C                -5.05941600   -1.30727900    4.01491800
 H                -5.65535600   -2.04949100    3.47644500
 H                -5.46853100   -0.31717500    3.79441500
 H                -5.20329700   -1.48832400    5.08688000
 C                -0.58930400    0.90825500    3.44377200
 H                -1.06620400    1.69005300    4.04066300
 H                -0.47483700    1.28755000    2.42132200
 H                 0.41813500    0.73543900    3.83099300
 C                 3.59460200   -1.49434300    0.47839100
 C                 3.33931200   -2.28245800   -0.65808100
 C                 4.21325800   -2.21545700   -1.74540900
 H                 4.00825400   -2.82952300   -2.62049600
 C                 5.34754100   -1.40884200   -1.72938300
 C                 5.60289700   -0.66634600   -0.57687400
 H                 6.48836800   -0.03483800   -0.53711800
 C                 4.75468100   -0.69488300    0.52901700
 C                 2.18656300   -3.24443300   -0.69976100
 H                 2.24660500   -3.88120600   -1.58554500
 H                 1.21936000   -2.72965400   -0.73758000
 H                 2.17392000   -3.88949700    0.18542800
 C                 6.25819300   -1.32120400   -2.92150200
 H                 6.16055300   -2.19915200   -3.56640300
 H                 7.30648500   -1.23576100   -2.61953000
 H                 6.02395400   -0.43910200   -3.52971700
 C                 5.09999100    0.12098300    1.74310400
 H                 5.56298100   -0.49069600    2.52631600
 H                 4.19891800    0.58194000    2.15328000
 H                 5.80835900    0.91184000    1.48283000
 C                 1.16082900    0.69869100    0.37957800
 C                 2.58783300    2.47861400    0.35141000
 C                 3.04041700    2.67850700   -0.97406800
 C                 3.41221500    3.96611000   -1.37001600
 H                 3.77097100    4.11331800   -2.38652200
 C                 3.33623800    5.04578200   -0.50175200
 H                 3.63905200    6.03635600   -0.82932100
 C                 2.87920400    4.84308800    0.79667200
 H                 2.81854100    5.67987000    1.48961800
 C                 2.49640700    3.57795200    1.23428000
 C                 3.11874700    1.54965300   -1.96116700
 H                 3.32006600    0.59750800   -1.46797300
 H                 3.90469700    1.73471300   -2.69985100
 H                 2.16826200    1.44579300   -2.50225100
 C                 1.98231000    3.37527800    2.62850100
 H                 0.90382600    3.18135500    2.61497800
 H                 2.15949000    4.26022900    3.24631800
 H                 2.45253100    2.50794000    3.10169800
```

4.1.7. $Ter_2N_3PAsCNDmpPC^tBu$

```
0 1
 As                0.77823100    0.67177600    1.27553800
 P                -1.33578200    0.17963200   -0.68141400
 P                -2.33391400    0.11104300    1.37243600
```

N	-0.07942600	1.33365800	-0.28278000
N	-0.25813700	-1.21698200	-0.50182000
N	1.33672200	-2.19083800	0.90715200
C	0.67621600	-1.17307500	0.51429500
C	-0.93327800	0.38936500	2.24662200
C	-0.26734000	2.74513400	-0.27560900
C	-0.47856100	-2.40957000	-1.27922500
C	-0.82704800	0.50770300	3.75702600
C	0.88433400	3.56618000	-0.11047300
C	-1.51419100	3.39513400	-0.46441900
C	-1.72808600	-3.06417700	-1.25402700
C	0.55295300	-2.92941800	-2.09496400
C	2.38427500	-2.07861200	1.83012400
C	-0.53859900	1.98066200	4.09223900
C	-2.11489100	0.07320800	4.45954100
C	0.33719800	-0.36225700	4.25312600
C	0.74699400	4.95293000	-0.01155800
C	2.30855000	3.10548100	-0.15851800
C	-1.59007000	4.79225800	-0.38342500
C	-2.81875200	2.75258200	-0.81705800
C	-1.91659800	-4.22299000	-2.01651800
C	-2.92022700	-2.63730400	-0.45954600
C	0.31927200	-4.09683900	-2.82849400
C	1.89100500	-2.30712400	-2.31848800
C	2.38879200	-2.98143000	2.91987000
C	3.47632000	-1.20296900	1.63911000
C	-0.48334100	5.58028900	-0.12728700
C	2.86070700	2.77667500	-1.41327600
C	3.16143400	3.25448700	0.95256200
C	-3.86367100	2.71557000	0.12812500
C	-3.08680100	2.41951200	-2.16364200
C	-0.90190100	-4.75138800	-2.79560800
C	-3.06171100	-3.06832900	0.87303100
C	-3.99029300	-1.98673400	-1.10384900
C	1.99776100	-1.19857500	-3.17856000
C	3.05377000	-2.94909600	-1.85348800
C	3.41613500	-2.91810600	3.85516100
C	1.29672500	-3.99907500	3.06874000
C	4.48488400	-1.17452100	2.60887500
C	3.65027100	-0.33971300	0.42145600
C	4.23782300	2.59916200	-1.53067000
C	1.98797900	2.67493000	-2.62889500
C	4.53761000	3.08396300	0.78598400
C	2.63825900	3.51561300	2.33646800
C	-5.12538900	2.26722800	-0.26867400
C	-3.68226200	3.22494800	1.53222900
C	-4.36528000	1.99917400	-2.51868700
C	-2.02731000	2.55882600	-3.21872000
C	-4.24998700	-2.79986200	1.55224600
C	-1.97342500	-3.85649100	1.54115000
C	-5.15301700	-1.72177700	-0.37964500
C	-3.91826400	-1.62078000	-2.56111300
C	3.26332600	-0.71402200	-3.50802000
C	0.77508200	-0.59934300	-3.81524700
C	4.29943000	-2.43435600	-2.21549900
C	2.98639800	-4.19430500	-1.01254800
C	4.45834000	-2.00575000	3.71715200
C	5.09903100	2.76113400	-0.44596000
C	-5.39891500	1.90882400	-1.58468600
C	-5.30150000	-2.11317500	0.94957100
C	4.42838000	-1.31257700	-3.03045600
C	6.58023300	2.55553700	-0.59245300
C	-6.78075000	1.49143200	-2.00374600
C	-6.54813500	-1.77854500	1.71792000
C	5.78140900	-0.75474700	-3.37168200
H	-0.40388900	2.10622300	5.17272400
H	0.36878400	2.33362500	3.59418000
H	-1.36838500	2.61880100	3.77361600
H	-2.00506300	0.17017800	5.54538300

H	-2.96511700	0.68921000	4.14884600
H	-2.35500900	-0.96992400	4.23017700
H	1.29243400	-0.06891600	3.80050700
H	0.44644700	-0.27240000	5.33944200
H	0.16552800	-1.41491600	4.01495900
H	1.65071500	5.54161800	0.11935500
H	-2.56313300	5.25151500	-0.53486500
H	-2.88718500	-4.70956100	-1.97244400
H	1.12431600	-4.47366700	-3.45305300
H	-0.56939900	6.66020300	-0.05898700
H	-1.06184300	-5.65456100	-3.37698200
H	3.40060300	-3.60433500	4.69857100
H	0.34116800	-3.54140100	3.34779600
H	1.12201600	-4.52746200	2.12622900
H	1.55329200	-4.73048000	3.83982100
H	5.31810300	-0.49003800	2.46441200
H	2.91269500	-0.53245200	-0.35857900
H	3.58918000	0.72047800	0.67791400
H	4.63895700	-0.51174400	-0.01494100
H	4.65153100	2.35829300	-2.50791400
H	1.27137800	1.85691700	-2.51429700
H	1.41152800	3.59375900	-2.78082900
H	2.58415700	2.49049700	-3.52569000
H	5.18678900	3.20880700	1.65101600
H	2.57187200	2.56559400	2.88164900
H	3.30702700	4.17317100	2.89955100
H	1.64222300	3.96174200	2.32793800
H	-5.92009200	2.21915200	0.47318100
H	-2.65225300	3.11763100	1.87555000
H	-3.93481300	4.29107000	1.59004800
H	-4.33822800	2.69217800	2.22628600
H	-4.56336700	1.74596500	-3.55832200
H	-1.62218000	3.57604600	-3.24026700
H	-1.18497900	1.88399500	-3.03256700
H	-2.42908600	2.32628600	-4.20811400
H	-4.35178900	-3.13443300	2.58280400
H	-0.99406600	-3.38999700	1.41046500
H	-2.16944700	-3.96426600	2.61142300
H	-1.89993200	-4.86252800	1.11056200
H	-5.97228200	-1.20768800	-0.87708300
H	-3.02413400	-1.03028900	-2.78214500
H	-3.88085900	-2.51539700	-3.19242000
H	-4.79124300	-1.03326900	-2.85435500
H	3.33949100	0.13715500	-4.18163000
H	0.07642900	-0.20577400	-3.06942400
H	1.04434700	0.21538300	-4.49207300
H	0.22563500	-1.35311600	-4.39008300
H	5.19535700	-2.93011400	-1.84624300
H	3.90998700	-4.32342200	-0.44188400
H	2.15029300	-4.14555300	-0.31184400
H	2.85447500	-5.08774500	-1.63466300
H	5.25452200	-1.96547000	4.45494100
H	7.14347200	3.19061400	0.09760900
H	6.85635500	1.51612500	-0.37520200
H	6.91699200	2.77653200	-1.60956500
H	-7.36098900	1.11821000	-1.15517500
H	-7.33340200	2.33669400	-2.43124900
H	-6.75140300	0.70873100	-2.76831500
H	-7.42039200	-1.71522300	1.06062700
H	-6.75669100	-2.52433800	2.49043300
H	-6.44400700	-0.80880000	2.21942000
H	5.78205600	-0.27694200	-4.35610600
H	6.08833200	0.00385000	-2.64084500
H	6.54804600	-1.53504900	-3.37259700

4.1.8. $Ter_2N_3PAsCNDmpPC^tBu$ – isomeric species

0 1

As	-1.73881100	0.46559200	-0.08619200

P	1.15820100	0.69984200	-0.36985300
P	-1.27695900	0.98592300	-2.30793300
N	-0.18902400	1.36749400	0.54090500
N	0.65413700	-0.99647000	-0.11939000
N	-1.18500700	-2.24827500	-0.91602700
C	-0.69092100	-1.18471300	-0.41335100
C	0.39639900	1.06429200	-2.09164000
C	-0.19369000	2.75499600	0.87218500
C	1.51155900	-2.03080700	0.38518400
C	1.26007400	1.49112300	-3.29592000
C	-1.27460600	3.63090500	0.56230900
C	0.91120100	3.31328500	1.57549400
C	2.84306000	-2.18342100	-0.07615300
C	1.05303800	-2.91248100	1.40194500
C	-2.52512900	-2.27557700	-1.36738500
C	1.20710200	0.39064800	-4.36910200
C	0.70359600	2.78097200	-3.92241900
C	2.71043000	1.76566900	-2.90257500
C	-1.16671700	5.00038200	0.82732700
C	-2.60673800	3.20133300	0.03649600
C	0.98088400	4.69431000	1.78502900
C	1.96509600	2.53973800	2.30521000
C	3.69536400	-3.10322200	0.54913400
C	3.48040400	-1.57264000	-1.28484300
C	1.95255700	-3.81528600	1.98255800
C	-0.31919800	-3.07369700	1.98883000
C	-2.68364000	-2.60455100	-2.73825000
C	-3.66731000	-2.06191300	-0.56713900
C	-0.03219400	5.55441600	1.39336000
C	-3.53928100	2.59877100	0.91028900
C	-2.99389500	3.50200500	-1.28499600
C	3.30690100	2.52421800	1.88849400
C	1.63316800	2.04636700	3.58495600
C	3.27576300	-3.90596800	1.59172700
C	3.15417600	-2.08781400	-2.55452800
C	4.61967000	-0.75614500	-1.14000800
C	-0.77603600	-2.22200900	3.00467100
C	-1.02617600	-4.26286400	1.70842200
C	-3.95729800	-2.64922400	-3.29163700
C	-1.48412500	-2.88995200	-3.59073500
C	-4.93053100	-2.12351300	-1.17081500
C	-3.62280700	-1.83324600	0.91634600
C	-4.76943800	2.18076400	0.41104700
C	-3.18725700	2.32309800	2.34244000
C	-4.23153700	3.05080600	-1.74770500
C	-2.13737200	4.34101900	-2.19104900
C	4.27050100	1.94483500	2.71880100
C	3.74539400	3.11467400	0.57841200
C	2.62000300	1.45114000	4.36766800
C	0.26088100	2.25960000	4.16018800
C	3.94451500	-1.74506700	-3.65253500
C	2.02900400	-3.06869100	-2.71991500
C	5.38371300	-0.44090800	-2.26451900
C	5.05188400	-0.25532300	0.20811700
C	-1.89160700	-2.59037900	3.76079400
C	-0.11727500	-0.90091500	3.24332600
C	-2.14462800	-4.58417200	2.47513800
C	-0.59270200	-5.18484500	0.60285700
C	-5.08750300	-2.40235800	-2.51715600
C	-5.11585700	2.35315200	-0.92906000
C	3.94751900	1.37615000	3.94685600
C	5.06326000	-0.92254600	-3.53204500
C	-2.58205800	-3.77488100	3.52412300
C	-6.39990100	1.79529400	-1.47135000
C	4.99391200	0.71024400	4.79479900
C	5.88074200	-0.54693500	-4.73531300
C	-3.78980000	-4.14888400	4.33682700
H	1.84902100	0.66018500	-5.21631900
H	1.55304700	-0.56574100	-3.97422600

H	0.18334800	0.26610100	-4.73648200
H	1.30964100	3.05829900	-4.79267400
H	-0.33086400	2.65135300	-4.25508800
H	0.73448200	3.61078500	-3.21008900
H	3.18798900	0.89492300	-2.45273900
H	3.28783700	2.04198300	-3.79208600
H	2.77278900	2.59944000	-2.19585100
H	-2.01590000	5.63032900	0.57671100
H	1.84842300	5.07833300	2.31517900
H	4.70570700	-3.19756400	0.16196900
H	1.57365800	-4.45517300	2.77428300
H	0.03935800	6.62245100	1.57358300
H	3.95311600	-4.60868000	2.06708100
H	-4.06132700	-2.88672700	-4.34735500
H	-0.82866400	-2.01599800	-3.66728200
H	-0.88219900	-3.69623500	-3.15991800
H	-1.78722900	-3.18007200	-4.60037500
H	-5.80523000	-1.95976400	-0.54483000
H	-2.61212800	-1.87987200	1.31638800
H	-4.03543600	-0.85079800	1.17219200
H	-4.21994000	-2.59439000	1.42815000
H	-5.46751700	1.68516300	1.08279100
H	-2.50122900	1.46617900	2.39983700
H	-2.68675400	3.17457600	2.81151700
H	-4.07751200	2.07342800	2.92506400
H	-4.50431200	3.24399100	-2.78295000
H	-2.31651300	4.09205800	-3.24010800
H	-2.37145300	5.40489500	-2.06007400
H	-1.07463200	4.21766200	-1.98018200
H	5.30876300	1.94703100	2.39240100
H	3.07568100	3.90845700	0.23987600
H	4.75561800	3.52750300	0.65716400
H	3.76100000	2.34541200	-0.20120300
H	2.35048700	1.06881400	5.35033400
H	0.05611800	3.33033200	4.27587800
H	-0.52210900	1.85582600	3.51589500
H	0.17346200	1.79064800	5.14374500
H	3.68737600	-2.15285000	-4.62839100
H	1.09826300	-2.71300700	-2.27037800
H	1.84521200	-3.27812700	-3.77718200
H	2.27205400	-4.01692400	-2.22528800
H	6.25799200	0.19562800	-2.14146900
H	4.19697200	0.01748400	0.83089500
H	5.61565700	-1.01759200	0.75846500
H	5.69985100	0.61960800	0.10531500
H	-2.22144300	-1.92858000	4.55959900
H	-0.38909500	-0.22298500	2.42579700
H	-0.44715700	-0.45914800	4.18574700
H	0.97418700	-0.96713300	3.25179500
H	-2.68148100	-5.50466600	2.25316300
H	-1.36181600	-5.93593600	0.40385500
H	-0.41568900	-4.61400900	-0.31391400
H	0.33647000	-5.71026900	0.84847200
H	-6.07861000	-2.44789700	-2.95941300
H	-6.74861600	2.35976200	-2.34058000
H	-6.25720300	0.75518800	-1.78832500
H	-7.19174500	1.80363600	-0.71653900
H	5.98333400	1.14491300	4.62526400
H	4.76163700	0.79706800	5.86026800
H	5.06317200	-0.35934800	4.56285600
H	5.87313800	-1.34081500	-5.48780800
H	5.48394800	0.35750400	-5.21249400
H	6.92079700	-0.34271800	-4.46534400
H	-3.78538000	-3.65229500	5.31119200
H	-4.71632400	-3.85861800	3.82602200
H	-3.83995100	-5.22882700	4.50558900

4.1.9. CNDmp

C	-2.32292600	-0.00252700	0.00001900

C	-1.62926700	-1.20780400	-0.00000600
C	-0.23599300	-1.23341300	-0.00002000
C	0.43456100	0.00047400	-0.00002800
C	-0.23866400	1.23290300	-0.00000800
C	-1.63188700	1.20424800	0.00002000
H	-3.40886300	-0.00371500	0.00003800
H	-2.17253000	-2.14864600	-0.00001800
H	-2.17719800	2.14390500	0.00003400
C	0.52935700	2.51985000	-0.00001000
H	-0.14747700	3.37687200	-0.00018200
H	1.17939300	2.59380500	0.87834300
H	1.17966000	2.59365200	-0.87817700
C	0.53490400	-2.51864000	-0.00000100
H	1.18560700	-2.59086200	-0.87799700
H	1.18487300	-2.59124600	0.87851900
H	-0.13998600	-3.37719400	-0.00044500
N	1.81517600	0.00195500	-0.00004200
C	2.99496300	0.00319900	0.00006300

4.2. Analysis of Cyclopentane-1,3-diyl derivatives

Table S2. Computational data for the cyclopentanediyls **2**.

	**NP**	**NAs**	**NSb**	**PAs**	**PSb**	**AsAs**
synthesis?	✓	✓	×	✓	×	×
E_{cbd} (**1**) [a.u.]	-2362.38075537	-4254.64879047	-2026.60465092	-4541.22621168	-2313.17811000	-6433.48904378
E_{cpd} (**2, s**) [a.u.]	-2765.06124362	-4657.31655534	-2429.24994477	-4943.88643318	-2715.82179945	-6836.14788714
E_{cpd} (**2, t**) [a.u.]	-2765.00454611	-4657.26602338	-2429.20469891	-4943.85769934	-2715.79410710	-6836.13099469
ΔE_{form} [kJ mol^{-1}] [a]	-148.0	-114.6	-55.6	-94.8	-51.4	-91.2
S–T gap [kJ mol^{-1}]	-148.9	-132.7	-118.8	-75.4	-72.7	-44.4
β(6,6) [b]	13%	11%	4%	24%	7%	38%
c_1	0.946	0.956	0.961	0.916	0.982	0.889
c_2	-0.250	-0.229	-0.132	-0.339	-0.185	-0.433
e p_z (E1)	1.29633	1.34073	1.45787	1.29790	1.44315	1.22819
e p_z (N1)	1.48432	1.45477	1.43699	1.58679	1.56992	1.56856
e p_z (E2)	1.17284	1.16374	1.13154	0.98872	0.92898	1.09205
e p_z (N2)	1.54945	1.54784	1.55258	1.63894	1.63915	1.63620
e p_z (C)	1.00870	1.00014	0.96801	0.98235	0.94515	0.97403
e p_z (N_{exo})	1.38723	1.37707	1.35582	1.37496	1.35444	1.38402
π e cpd-ring (NBO)	6.51	6.51	6.55	6.49	6.53	6.50
E–E BO (WBI)	0.3129	0.2875	0.2615	0.3889	0.3432	0.4337
λ_{max} calc.	476	518	542	674	715	750
HOMO	–0.17495	–0.17161	–0.16343	–0.15961	–0.15204	–0.15380
LUMO	–0.05435	–0.05990	–0.05810	–0.07163	–0.07009	–0.07582
NICS(0) [ppm]	–8.32	–7.45	–6.40	–5.62	–4.84	–4.66
NICS(1) [ppm]	–6.65	–6.10	–5.51	–3.24	–3.18	–2.90

[a] E(CNDmp): -402.624107815 a.u.; [b]; [c];

Frontier Orbitals

LUMO

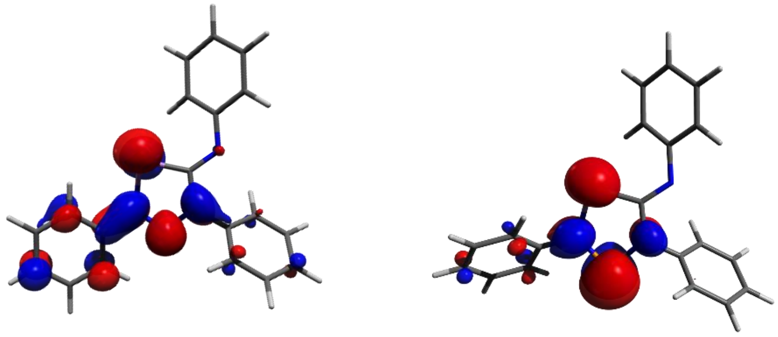

HOMO

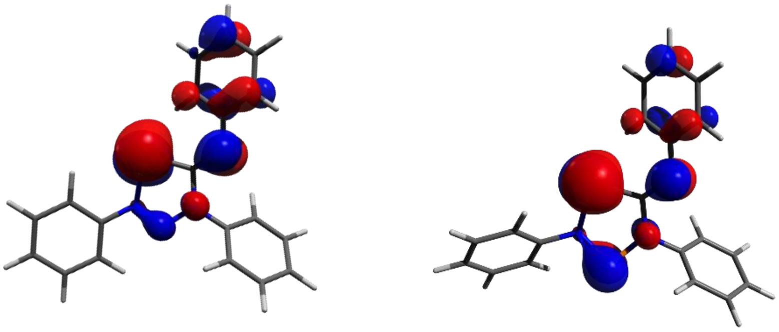

Table S3. Computated energies and energy differences for the formation of the cyclopentanediyls **2** (red: accessible, grey: no formation observed, green: E2 lighter than E1, formation not observed, blue: not attempted in lieu of suitable precursors).

**E2 =**	**P**	**As**	**Sb**
**E1 =**	**N**	**N**	**N**
4ring	**-2362.38075537**	**-4254.64879047**	**-2026.60465092**
intermediate	**-2764.97362419**	**-4657.24897956**	**-2429.18047044**
5ring	**-2765.06124362**	**-4657.31655534**	**-2429.24994477**
ΔE 4ring-int	**82.0**	**62.8**	**126.8**
ΔE int-5ring	-230.0	-177.4	-182.4
ΔE 4ring-5ring	**-148.0**	**-114.6**	**-55.6**

**E2 =**	N	**P**	**As**	**Sb**
**E1 =**	P	**P**	**P**	**P**
4ring	-2362.38075537	**-2648.95984829**	**-4541.22621168**	**-2313.17811000**
intermediate	**-2764.97362419**	**-3051.60297046**	**-4943.87650537**	**-2715.81322120**
5ring	-2765.09987684	**-3051.62831340**	**-4943.88825952**	**-2715.81589049**
ΔE 4ring-int	**82.0**	**-49.9**	**-68.8**	**-28.9**
ΔE int-5ring	-331.5	-66.5	-30.9	-7.0
ΔE 4ring-5ring	**-249.5**	**-116.5**	**-99.6**	**-35.9**

**E2 =**	N	P	**As**	Sb
**E1 =**	As	As	**As**	As
4ring	-4254.64879047	-4541.22621168	**-6433.48904378**	-4205.05946650
intermediate	**-4657.24897956**	**-4943.87650537**	**-6836.14264078**	-4607.70112786
5ring	-4657.36155483	-4943.88643325	**-6836.14788719**	-4607.70330624
ΔE 4ring-int	**62.8**	**-68.8**	**-77.4**	**-46.1**
ΔE int-5ring	-295.6	-26.1	-13.8	-5.7
ΔE 4ring-5ring	**-232.8**	**-94.8**	**-91.2**	**-51.8**

**E2 =**	N	P	As
**E1 =**	Sb	Sb	Sb
4ring	-2026.60465092	-2313.17811000	-4205.05946650
intermediate	**-2429.18047044**	**-2715.81322120**	-4607.70112786
5ring	-2429.29010829	-2715.82179943	-4607.70445476
ΔE 4ring-int	**126.8**	**-28.9**	**-46.1**
ΔE int-5ring	-287.9	-22.5	-8.7
ΔE 4ring-5ring	**-161.1**	**-51.4**	**-54.8**

5. References

[1] T. Beweries, R. Kuzora, U. Rosenthal, A. Schulz, A. Villinger, *Angew. Chem. Int. Ed.* **2011**, *50*, 8974–8978.

[2] S. Demeshko, C. Godemann, R. Kuzora, A. Schulz, A. Villinger, *Angew. Chem. Int. Ed.* **2013**, *52*, 2105–2108.

[3] A. Hinz, A. Schulz, A. Villinger, J.-M. Wolter, *J. Am. Chem. Soc.* **2015**, *137*, 3975–3980.

[4] A. Hinz, J. Rothe, A. Schulz, A. Villinger, *Dalton Trans.* **2015**, *2*, 10.1039/C5DT02711J.

[5] W. P. Weber, G. W. Gokel, *Tetrahedron Lett.* **1972**, *13*, 1637–1640.

[6] G. R. Fulmer, A. J. M. Miller, N. H. Sherden, H. E. Gottlieb, A. Nudelman, B. M. Stoltz, J. E. Bercaw, K. I. Goldberg, *Organometallics* **2010**, *29*, 2176–2179.

[7] G. M. Sheldrick, **2013**, SHELXS–2013.

[8] G. M. Sheldrick, **2013**, SHELXL–2013.

[9] G. M. Sheldrick, **2004**, SADABS 2.

[10] M. J. Frisch, G. W. Trucks, H. B. Schlegel, G. E. Scuseria, M. A. Robb, J. R. Cheeseman, G. Scalmani, V. Barone, B. Mennucci, G. A. Petersson, H. Nakatsuji, M. Caricato, X. Li, H. P. Hratchian, A. F. Izmaylov, J. Bloino, G. Zheng, J. L. Sonnenberg, M. Hada, M. Ehara, K. Toyota, R. Fukuda, J. Hasegawa, M. Ishida, T. Nakajima, Y. Honda, O. Kitao, H. Nakai, T. Vreven, J. A. Montgomery, J. E. Peralta, F. Ogliaro, M. Bearpark, J. J. Heyd, E. Brothers, K. N. Kudin, V. N. Staroverov, R. Kobayashi, J. Normand, K. Raghavachari, A. Rendell, J. C. Burant, S. S. Iyengar, J. Tomasi, M. Cossi, N. Rega, J. M. Millam, M. Klene, J. E. Knox, J. B. Cross, V. Bakken, C. Adamo, J. Jaramillo, R. Gomperts, R. E. Stratmann, O. Yazyev, A. J. Austin, R. Cammi, C. Pomelli, J. W. Ochterski, R. L. Martin, K. Morokuma, V. G. Zakrzewski, G. A. Voth, P. Salvador, J. J. Dannenberg, S. Dapprich, A. D. Daniels, Ö. Farkas, J. B. Foresman, J. V. Ortiz, J. Cioslowski and D. J. Fox, *Gaussian 09*, Gaussian, Inc., Wallingford CT, **2009**.

[11] T. Lu, F. Chen, *J. Comput. Chem.* **2012**, *33*, 580–592.

[12] E. D. Glendening, C. R. Landis, F. Weinhold, *J. Comput. Chem.* **2013**, *34*, 1429–1437.

[13] E. D. Glendening, F. Weinhold, *J. Comput. Chem.* **1998**, *19*, 593–609.

[14] E. D. Glendening, F. Weinhold, *J. Comput. Chem.* **1998**, *19*, 610–627.
